# Phenotype-based targeted treatment of SGLT2 inhibitors and GLP-1 receptor agonists in type 2 diabetes

**DOI:** 10.1007/s00125-024-06099-3

**Published:** 2024-02-22

**Authors:** Pedro Cardoso, Katie G. Young, Anand T. N. Nair, Rhian Hopkins, Andrew P. McGovern, Eram Haider, Piyumanga Karunaratne, Louise Donnelly, Bilal A. Mateen, Naveed Sattar, Rury R. Holman, Jack Bowden, Andrew T. Hattersley, Ewan R. Pearson, Angus G. Jones, Beverley M. Shields, Trevelyan J. McKinley, John M. Dennis, on behalf of the MASTERMIND consortium

**Affiliations:** 1 Institute of Biomedical & Clinical Science, University of Exeter Medical School, Exeter, UK; 2 Division of Molecular & Clinical Medicine, Ninewells Hospital and Medical School, University of Dundee, Dundee, UK; 3 Institute of Health Informatics, University College London, London, UK; 4 Institute of Cardiovascular and Medical Sciences, University of Glasgow, Glasgow, UK; 5 Diabetes Trials Unit, Oxford Centre for Diabetes, Endocrinology and Metabolism, University of Oxford, Oxford, UK; 6 Oxford NIHR Biomedical Research Centre, Churchill Hospital, Oxford, UK

**Keywords:** Bayesian non-parametric modelling, GLP1-receptor agonists, Heterogeneous treatment effects, Precision medicine, SGLT2-inhibitors, Type 2 diabetes

## Abstract

**Aims/hypothesis:**

A precision medicine approach in type 2 diabetes could enhance targeting specific glucose-lowering therapies to individual patients most likely to benefit. We aimed to use the recently developed Bayesian causal forest (BCF) method to develop and validate an individualised treatment selection algorithm for two major type 2 diabetes drug classes, sodium–glucose cotransporter 2 inhibitors (SGLT2i) and glucagon-like peptide-1 receptor agonists (GLP1-RA).

**Methods:**

We designed a predictive algorithm using BCF to estimate individual-level conditional average treatment effects for 12-month glycaemic outcome (HbA_1c_) between SGLT2i and GLP1-RA, based on routine clinical features of 46,394 people with type 2 diabetes in primary care in England (Clinical Practice Research Datalink; 27,319 for model development, 19,075 for hold-out validation), with additional external validation in 2252 people with type 2 diabetes from Scotland (SCI-Diabetes [Tayside & Fife]). Differences in glycaemic outcome with GLP1-RA by sex seen in clinical data were replicated in clinical trial data (HARMONY programme: liraglutide [*n*=389] and albiglutide [*n*=1682]). As secondary outcomes, we evaluated the impacts of targeting therapy based on glycaemic response on weight change, tolerability and longer-term risk of new-onset microvascular complications, macrovascular complications and adverse kidney events.

**Results:**

Model development identified marked heterogeneity in glycaemic response, with 4787 (17.5%) of the development cohort having a predicted HbA_1c_ benefit >3 mmol/mol (>0.3%) with SGLT2i over GLP1-RA and 5551 (20.3%) having a predicted HbA_1c_ benefit >3 mmol/mol with GLP1-RA over SGLT2i. Calibration was good in hold-back validation, and external validation in an independent Scottish dataset identified clear differences in glycaemic outcomes between those predicted to benefit from each therapy. Sex, with women markedly more responsive to GLP1-RA, was identified as a major treatment effect modifier in both the UK observational datasets and in clinical trial data: HARMONY-7 liraglutide (GLP1-RA): 4.4 mmol/mol (95% credible interval [95% CrI] 2.2, 6.3) (0.4% [95% CrI 0.2, 0.6]) greater response in women than men. Targeting the two therapies based on predicted glycaemic response was also associated with improvements in short-term tolerability and long-term risk of new-onset microvascular complications.

**Conclusions/interpretation:**

Precision medicine approaches can facilitate effective individualised treatment choice between SGLT2i and GLP1-RA therapies, and the use of routinely collected clinical features for treatment selection could support low-cost deployment in many countries.

**Graphical Abstract:**

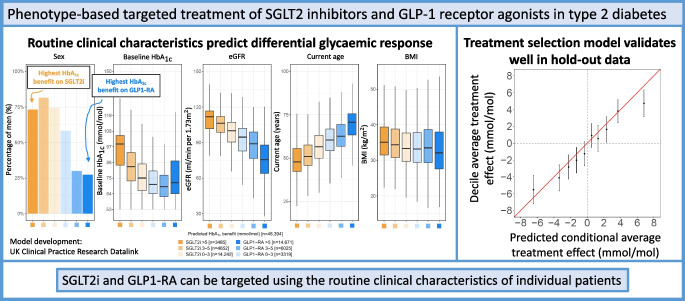

**Supplementary Information:**

The online version contains peer-reviewed but unedited supplementary material available at 10.1007/s00125-024-06099-3.


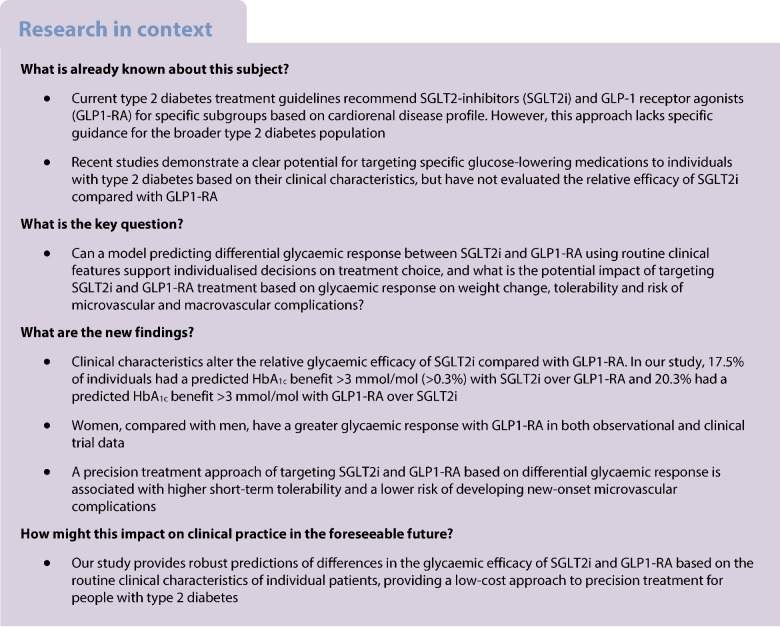


## Introduction

A precision medicine approach in type 2 diabetes would aim to target specific glucose-lowering therapies to individual patients most likely to benefit [1]. Current stratification in type 2 diabetes treatment guidelines involves preferential prescribing of two major drug classes, sodium–glucose cotransporter 2 inhibitors (SGLT2i) and glucagon-like peptide-1 receptor agonists (GLP1-RA), to subgroups of people with or at high risk of cardiorenal disease [2]. Evidence informing these recommendations comes from average treatment effect (ATE) estimates derived from placebo-controlled cardiovascular and renal outcome trials, which have predominantly recruited participants with advanced atherosclerotic cardiovascular risk or established cardiovascular disease [3, 4]. Consequently, there is limited evidence on the benefits of SGLT2i and GLP1-RA for individuals in the broader type 2 diabetes population and, given the lack of head-to-head trials, of the relative efficacy of the two drug classes for individual patients.

Recent studies have demonstrated a clear potential for a precision medicine approach based on glycaemic response, with the TRIMASTER crossover trial establishing a greater efficacy of SGLT2i compared with DPP4 inhibitors (DPP4i) in those with better renal function, and a greater efficacy of thiazolidinedione therapy compared with DPP4i in those with obesity (BMI > 30 kg/m^2^) compared to those without obesity [5]. Given these findings, a trial-data-validated prediction model to support individualised treatment selection has recently been developed for SGLT2i vs DPP4i therapy [6]. For GLP1-RA, although recent studies have identified robust heterogeneity in treatment response based on pharmacogenetic markers and markers of insulin secretion [7, 8], the influence of these markers on relative differences in clinical outcomes compared with other drug classes, and therefore their utility for targeting treatment, has not previously been assessed.

Given the lack of evidence to support targeted treatment of SGLT2i compared with GLP1-RA therapies, we aimed to develop and validate a prediction model to provide individual patient-level estimates of differences in 12-month glycaemic (HbA_1c_) outcomes for the two drug classes based on routinely collected clinical features. We also evaluated the downstream impacts of targeting therapy based on glycaemic response on secondary outcomes of weight change, tolerability and longer-term risk of new-onset microvascular complications, macrovascular complications and adverse kidney events.

## Methods

### Study population

**Fig. 1:**
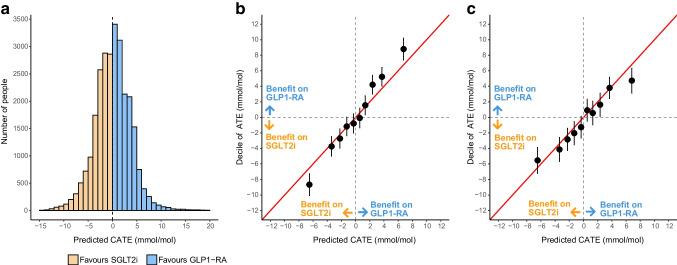
Predicted CATE effects and model calibration. (**a**) Distribution of CATE estimates for SGLT2i vs GLP1-RA in the CPRD development cohort; negative values reflect a predicted HbA_1c_ treatment benefit on SGLT2i and positive values reflect a predicted treatment benefit on GLP1-RA. (**b**) Calibration between ATE and predicted CATE estimates, by decile of predicted CATE in the development cohort. (**c**) Calibration of CATE estimates in the validation cohort. ATE estimates are adjusted for all the variables used in the treatment selection model (see Methods)

Individuals with type 2 diabetes initiating SGLT2i and GLP1-RA therapies between 1 January 2013 and 31 October 2020 were identified in the UK population-representative Clinical Practice Research Datalink (CPRD) Aurum dataset [9], following our previously published cohort profile [10] (see https://github.com/Exeter-Diabetes/CPRD-Codelists for all codelists). We excluded individuals prescribed either therapy as first-line treatment (not recommended in UK guidelines) [11], co-treated with insulin, and with a diagnosis of end-stage renal disease (ESRD) (electronic supplementary material [ESM] Fig. 1). Owing to low numbers, we also excluded individuals initiating the GLP1-RA semaglutide (*n*=784 study-eligible individuals with outcome HbA_1c_ recorded) [12]. The final CPRD cohort was randomly split 60:40 into development and hold-back validation sets, maintaining the proportion of individuals receiving SGLT2i and GLP1-RA in each set. For model development, individuals were excluded from the development and validation sets if they initiated multiple glucose-lowering treatments on the same day; their therapies were initiated less than 61 days since the start of a previous therapy; their baseline HbA_1c_ was <53 mmol/mol (7%); they had a missing baseline HbA_1c_; or they had a missing outcome HbA_1c_ (Table 1, ESM Fig. 1).

**Table 1:** Baseline clinical characteristics of patients initiating GLP1-RA and SGLT2i from the CPRD

Baseline characteristic	GLP1-RA (*n*=28,081)		SGLT2i (*n*=84,193)		SMD
		Missing (%)		Missing (%)	
Current age, years	57.7 [11.2]		58.4 [10.8]		0.064
Duration of diabetes, years	9.4 [6.4]		9.4 [6.6]		0.012
Year of drug start	2017 [2]		2017 [2]		0.390
Sex					0.155
Male	14,960 (53.3)		51,288 (60.9)		
Female	13,121 (46.7)		32,905 (39.1)		
Ethnicity					0.255
White	24,063 (85.7)		64,111 (76.1)		
South Asian	2144 (7.6)		12,234 (14.5)		
Black	987 (3.5)		3861 (4.6)		
Other	262 (0.9)		1316 (1.6)		
Mixed	246 (0.9)		885 (1.1)		
Missing	379 (1.3)		1786 (2.1)		
SGLT2i type					
Canagliflozin			14,965 (17.8)		
Dapagliflozin			36,250 (43.1)		
Empagliflozin			32,860 (39.0)		
Ertugliflozin			137 (0.2)		
GLP1-RA type					
Dulaglutide	10,337 (36.8)				
Exenatide (short-acting)	1367 (4.9)				
Exenatide (long-acting)	2377 (8.5)				
Liraglutide	11,260 (40.1)				
Lixisenatide	2751 (9.8)				
Index of multiple deprivation		18 (0.1)		47 (0.1)	0.033
1 (Least deprived)	4568 (16.3)		14,143 (16.8)		
2	4925 (17.5)		14,836 (17.6)		
3	5393 (19.2)		16,194 (19.2)		
4	6099 (21.7)		18,841 (22.4)		
5 (Most deprived)	7078 (25.2)		20,132 (23.9)		
Smoking status					0.048
Active	4689 (16.7)		14,265 (16.9)		
Ex-smoker	15,543 (55.4)		45,283 (53.8)		
Non-smoker	6605 (23.5)		21,311 (25.3)		
Missing	1244 (4.4)		3334 (4.0)		
Number of glucose-lowering drug classes ever prescribed					0.420
2	2886 (10.3)		18,724 (22.2)		
3	6772 (24.1)		25,570 (30.4)		
4	10,536 (37.5)		25,217 (30.0)		
≥5	7887 (28.1)		14,682 (17.4)		
Number of other current glucose-lowering drugs					0.137
0	1161 (4.1)		5155 (6.1)		
1	9948 (35.4)		34,032 (40.4)		
2	12,422 (44.2)		35,540 (42.2)		
3	3862 (13.8)		9187 (10.9)		
≥4	233 (0.8)		279 (0.3)		
Background therapy					
Metformin	24,075 (85.7)		73,392 (87.2)		0.042
Sulfonylurea	13,312 (47.4)		30,165 (35.8)		0.237
DPP4i	4595 (16.4)		23,256 (27.6)		0.274
SGLT2i	4019 (14.3)				–
Thiazolidinedione	1312 (4.7)		2374 (2.8)		0.098
GLP1-RA			4603 (5.5)		–
Biomarkers					
HbA_1c_, mmol/mol^a^	78.6 [17.1]	5795 (20.6)	76.9 [16.9]	10,252 (12.2)	0.101
HbA_1c_, %^a^	9.3 [3.7]	5795 (20.6)	9.2 [3.7]	10,252 (12.2)	0.101
BMI, kg/m^2^	37.3 [7.2]	771 (2.7)	33.7 [6.9]	3234 (3.8)	0.522
eGFR, ml/min per 1.73 m^2^	92.0 [19.7]	68 (0.2)	94.7 [15.5]	217 (0.3)	0.152
HDL-cholesterol, mmol/l	1.1 [0.3]	1333 (4.7)	1.1 [0.3]	3019 (3.6)	0.083
ALT, IU/l	35.1 [20.6]	1801 (6.4)	34.5 [20.2]	4841 (5.7)	0.027
Albumin, g/l	41.6 [3.9]	1347 (4.8)	42.0 [3.9]	3667 (4.4)	0.109
Bilirubin, µmol/l	9.1 [4.7]	1187 (4.2)	9.5 [5.0]	3385 (4.0)	0.084
Total cholesterol, mmol/l	4.4 [1.1]	167 (0.6)	4.3 [1.1]	398 (0.5)	0.039
Mean arterial BP, mmHg	96.1 [9.0]	82 (0.3)	96.2 [9.0]	270 (0.3)	0.016
Microvascular complications					
Nephropathy	730 (2.6)		1623 (1.9)		0.045
Neuropathy	7942 (28.3)		20,161 (23.9)		0.099
Retinopathy	10,540 (37.5)		31,664 (37.6)		0.002
Cardiovascular conditions					
Angina	3224 (11.5)		8251 (9.8)		0.055
Atherosclerotic CVD^a^	6285 (22.4)		16,530 (19.6)		0.068
Atrial fibrillation	1737 (6.2)		4129 (4.9)		0.056
Cardiac revascularisation	1863 (6.6)		5632 (6.7)		0.002
Heart failure	1662 (5.9)		3654 (4.3)		0.072
Hypertension	16,833 (59.9)		46,550 (55.3)		0.094
Ischaemic heart disease	4181 (14.9)		11,309 (13.4)		0.042
Myocardial infarction	1943 (6.9)		5655 (6.7)		0.008
Peripheral arterial disease	1703 (6.1)		3836 (4.6)		0.067
Stroke	1270 (4.5)		3422 (4.1)		0.023
Transient ischaemic attack	766 (2.7)		2126 (2.5)		0.013
Other conditions					
CKD	2684 (9.6)		2962 (3.5)		0.246
Chronic liver disease	3754 (13.4)		10,241 (12.2)		0.036
QRISK2 10-year score	23.5 [13.5]	1573 (5.6)	23.5 [13.2]	6215 (7.4)	0.006
HbA_1c_ outcome					
HbA_1c_, mmol/mol	66.3 [18.3]	15,397 (54.8)	64.2 [15.0]	40,025 (47.5)	0.126
HbA_1c_, %	8.2 [3.8]	15,397 (54.8)	8.0 [3.5]	40,025 (47.5)	0.126
Month of HbA_1c_ measure	8.9 [3.5]	15,397 (54.8)	9.2 [3.5]	40,025 (47.5)	0.071

Data are mean [SD] and number (%)

SMD: Standardised mean difference (≥0.1 is a common metric for a meaningful imbalance between treatment groups). Atherosclerotic cardiovascular disease: composite of myocardial infarction, stroke, ischaemic heart disease, peripheral arterial disease and revascularisation

^a^Closest values to treatment start in the previous 6 months

### Additional cohorts

The same eligibility criteria were applied to define an independent cohort in Scotland for model validation (SCI-Diabetes [Tayside & Fife], containing longitudinal observational data including biochemical investigations and prescriptions). To assess reproducibility of differences in HbA_1c_ response by sex with GLP1-RA therapy, we accessed individual-level data on participants initiating the GLP1-RAs albiglutide and liraglutide in the HARMONY clinical trial programme (sponsored by GlaxoSmithKline [GSK]), an international randomised placebo-controlled trial designed to evaluate the cardiovascular benefit of albiglutide with type 2 diabetes [13], and the Predicting Response to Incretin Based Agents (PRIBA) prospective cohort study (UK 2011–2013) [14], designed to test whether individuals with low insulin secretion have lesser glycaemic response to incretin-based treatments.

### Outcomes

The primary outcome was achieved HbA_1c_ at 12 months post drug initiation on unchanged glucose-lowering therapy. Given the variability in the timing of follow-up testing in UK primary care, this outcome was defined as the closest eligible HbA_1c_ value to 12 months (within 3–15 months) after initiation. To allow for potential differential effects of follow-up duration on HbA_1c_, we included an additional covariate to capture the month the outcome HbA_1c_ was recorded.

Secondary outcomes comprised short-term 12 month weight change after initiation (closest recorded weight to 12 months, within 3–15 months), and, as a proxy for drug tolerability, treatment discontinuation within 6 months of drug initiation (as such short-term discontinuation is unlikely to be related to a lack of glycaemic response), and longer-term outcomes up to 5 years after initiation: new-onset major adverse cardiovascular events (MACE: composite of myocardial infarction, stroke and cardiovascular death); new-onset heart failure; new-onset adverse kidney outcome (a drop of ≥40% in eGFR from baseline or reaching chronic kidney disease [CKD] stage 5 [7]); and new-onset microvascular complications (ESM Fig. 2). We focused on only new-onset cardiorenal events (excluding individuals with pre-existing conditions of interest), as those with pre-existing disease have a clear indication for SGLT2i and GLP1-RA in current guidelines irrespective of differences in glycaemic outcome.

### Predictors

Candidate predictors were selected to represent readily available (available in >75% of individuals) routine clinical features and comprised current age, duration of diabetes, year of therapy start, sex (self-reported), ethnicity (self-reported, categorised into major UK groups: White, South Asian, Black, Mixed, other), social deprivation (index of multiple deprivation quintile), smoking status, the number of current, and ever, prescribed glucose-lowering drug classes, baseline HbA_1c_ (closest to treatment start date; range in previous 6 months to +7 days), clinical parameters: BMI, eGFR (CKD-EPI formula [15]), HDL-cholesterol, alanine aminotransferase (ALT), albumin, bilirubin, total cholesterol and mean arterial blood pressure (all defined as closest values to treatment start in the previous two years), microvascular complications: nephropathy, neuropathy, retinopathy, and major comorbidities: angina, atherosclerotic cardiovascular disease, atrial fibrillation, cardiac revascularisation, heart failure, hypertension, ischaemic heart disease, myocardial infarction, peripheral arterial disease, stroke, transient ischaemic attack, CKD and chronic liver disease.

### Treatment selection model development

We used the recently proposed Bayesian causal forest (BCF) structure, a framework specifically designed to estimate heterogeneous treatment effects (henceforth: conditional average treatment effects [CATEs]) [16, 17] (ESM Methods: Model overview). The CATE for an individual is conditional on their clinical characteristics, and represents the predicted differential effects of the two drug classes on HbA_1c_ outcome . The BCF framework also minimises confounding from indication bias and allows for flexibility in defining model structure and outputs, and is an extension of Bayesian additive regression tree (BART) counterfactual models [18]. The model development process consisted of a first step of propensity score estimation to minimise confounding due to prescribing by indication [19], (ESM Methods: Propensity score estimation), and a second step of model development, using the R packages *bcf* (version 2.0.1) [17] and *sparseBCF* (version 1.0) [19] packages. Variable selection, based on each variable’s splitting probabilities, was deployed to develop a parsimonious final model whilst maintaining predictive accuracy (ESM Methods: Variable selection). The propensity score was not included in the final predictor set as it did not meet our threshold for variable selection (ESM Methods: Final model fit); however, as a sensitivity analysis, we refitted the final model, including the propensity score in the predictor set and compared predictions across the two models. Currently, the standard BCF software cannot account for missing data [20], so we used a complete case analysis, informed by our previous study showing a limited impact of missing data on predicting CATE in a similar primary care dataset [21]. To evaluate the degree of model-predicted treatment effect heterogeneity, differential HbA_1c_ response—the difference in achieved HbA_1c_ between drug classes—was extracted from the final model for all individuals.

Variable importance was estimated based on best linear projection (ESM Methods: Variable importance). To assess how CATE estimates varied across major routine clinical features, we also summarised the marginal distributions of key predictor variables (sex, baseline HbA_1c_, eGFR, current age and BMI) across subgroups defined by the degree of predicted glycaemic differences (SGLT2i benefit of 0–3, 3–5 or >5 mmol/mol [0–0.3, 0.3–0.5 or >0.5%]; GLP1-RA benefit of 0–3, 3–5 or >5 mmol/mol).

### Model validation

Evaluating the accuracy of predicted CATE is a significant challenge since, in practice, true CATE estimates are unobserved as a single individual receives only one therapy, meaning the counterfactual outcome they would have had on the alternative therapy is unobserved [22]. As such, to validate predicted CATE estimates, we first split validation sets into subgroups based on predicted CATE estimates and then compared the average CATE estimate within each subgroup to estimates derived from a set of alternative models fitted to each of the subgroups in turn. These latter models target the average treatment effect (ATE) within a population of individuals (rather than the conditional average treatment effect [CATE]), with desirable properties justified in the literature [23]. This validation framework further develops the concordant–discordant approach previously proposed in Dennis et al [6]. If the average CATE estimates in each subgroup (from the BCF model) align with the ATE estimates from the alternative models, this provides evidence that ATEs are consistent across different inference methods within each subgroup. Restricting the ATE estimates for each subgroup allows for simpler comparison ATE models to be used, since the distribution of covariates in each subgroup is expected to be more consistent within each subgroup than for the complete data. For validation, subgroups were defined by decile of predicted CATE in CPRD and, owing to the smaller cohort size, by quintile in Tayside & Fife.

To estimate the ATEs within subgroups, we used regression adjustment as the primary approach, estimating the ATE as the average difference in HbA_1c_ outcome between individuals receiving each therapy class within each subgroup Bayesian linear regression, adjusting for the full covariate set used in the HbA_1c_ treatment selection model (full covariate set; Table 2), with all continuous predictors included as 3-knot restricted cubic splines [6]. As a sensitivity analysis, we estimated CATE using propensity score matching with and without regression adjustment (ESM Methods).

**Table 2:** Baseline clinical features included in the treatment selection algorithm after variable selection

Features predictive of HbA_1c_ outcome with SGLT2i	Features predictive of differential HbA_1c_ outcome with GLP1-RA compared with SGLT2i
Current age	Current age
Duration of diabetes	Sex
Number of glucose-lowering drug classes ever prescribed	Number of other current glucose-lowering drugs
Number of other current glucose-lowering drugs	Baseline HbA_1c_
Baseline HbA_1c_	BMI
eGFR	eGFR
ALT	Heart failure
Peripheral arterial disease	Ischaemic heart disease
	Neuropathy
	Peripheral arterial disease
	Retinopathy

The BCF treatment selection model is structured into two parts: a part that identifies features predictive of HbA_1c_ outcome with SGLT2i, and a part that identifies differential HbA_1c_ outcome with GLP1-RA compared with SGLT2i. HbA_1c_ outcome with SGLT2i is estimated using the set of features on the left of the table, HbA_1c_ outcome with GLP1-RA is estimated using both sets of features in the table

As our overall dataset predominantly included individuals of white ethnicity, we assessed the accuracy of predicted HbA_1c_ treatment effects in a subgroup of individuals of South Asian, Black, Other and Mixed ethnicity. We also evaluated accuracy of predicted HbA_1c_ treatment effects in those with and without cardiovascular disease. We also evaluated the reproducibility of observed differences in HbA_1c_ response by sex in participants receiving GLP1-RA in the HARMONY clinical trial, the PRIBA prospective study, and Tayside & Fife.

### Secondary outcomes

Specific cohorts were defined to evaluate each secondary outcome to mitigate selection bias and maximise the number of individuals available for analysis (ESM Fig. 2; ESM Methods: Secondary outcomes). All cohorts required complete predictor data for the HbA_1c_-based treatment selection model. To evaluate treatment effect heterogeneities, subgroups were defined by the degree of predicted glycaemic differences (SGLT2i benefit of 0–3, 3–5 or >5 mmol/mol [0–0.3, 0.3–0.5 or >0.5%]; GLP1-RA benefit of 0–3, 3–5 or >5 mmol/mol). As for validation of differences in HbA_1c_ outcomes, we evaluated subgroup-level ATEs using regression adjustment as the primary approach, with propensity score matching with and without regression adjustment deployed as sensitivity analysis. For evaluation of new-onset cardiovascular and renal outcomes, the propensity score model was refitted incorporating baseline cardiovascular risk as an additional predictor (QRISK2 predicted probability of new-onset myocardial infarction or stroke [24]). Absolute HbA_1c_ response was evaluated by drug class as adjusted (full covariate set) HbA_1c_ change from baseline using Bayesian linear regression. To evaluate differences by drug class in 12 month weight change, we included all individuals with a recorded baseline weight (closest value to 2 years prior to treatment initiation) and a valid outcome weight. Treatment effects were estimated using an adjusted (full covariate set) Bayesian linear regression model with an interaction between the received treatment and the predicted HbA_1c_ treatment benefit subgroup, with adjustment for baseline weight. Similarly, differences in treatment discontinuation were estimated using adjusted (full covariate set) Bayesian logistic regression with a treatment-by-HbA_1c_ benefit subgroup interaction.

For longer-term outcomes, we included only individuals without the outcome of interest at therapy initiation, thus evaluating only incident events. Individuals were followed for up to 5 years using an intention-to-treat approach from the date of therapy initiation until the earliest of: the outcome of interest, the date of general practitioner (GP) practice deregistration or death, or the end of the study period. For each outcome, adjusted (full covariate set) Bayesian Cox proportional hazards models with treatment-by-HbA_1c_ benefit subgroup interactions were fitted with additional adjustment for QRISK2 predicted probability of new-onset myocardial infarction or stroke.

All analyses were conducted using R (version 4.1.2; R Foundation for Statistical Computing, Austria). We followed TRIPOD prediction model reporting guidance (ESM Materials) [25].

## Results

We included 84,193 people with type 2 diabetes initiating SGLT2i and 28,081 initiating GLP1-RA (ESM Fig. 1). The mean age of individuals was 58.2 (SD=10.9) years, 66,248 (59%) were men, and 88,174 (79%) were of white ethnicity. Baseline clinical characteristics by initiated drug class are reported in Table 1.

### Model development

For the development of the 12 month HbA_1c_ response treatment selection model, individuals with a measured HbA_1c_ outcome were randomly split 60:40 into development (*n*=31,346) and validation (*n*=20,865) cohorts (ESM Fig. 1; Baseline characteristics by cohort: ESM Table 1). Mean unadjusted 12 month HbA_1c_ response (change from baseline in HbA_1c_) was −12.0 (SD 15.3) mmol/mol (−1.1% [SD 1.4%]) for SGLT2i and −11.7 (SD 17.6) mmol/mol (−1.1% [SD 1.6%]) for GLP1-RA.

After variable selection [26] (ESM Fig. 3), we identified multiple clinical factors predictive of HbA_1c_ response with SGLT2i (the reference drug class in the model), and multiple factors predictive of differential HbA_1c_ response with GLP1-RA compared with SGLT2i therapy (Table 2). The final BCF model was fitted to 27,319 (87.2% of the starting development cohort) individuals with complete data for all selected clinical factors. In sensitivity analysis, the model predictions for final BCF model were similar to the BCF model with the full covariate set (ESM Fig. 4). Overall model fit and performance statistics for predicting achieved HbA_1c_ outcome in internal validation for both the development and hold-out cohorts are reported in ESM Table 2. The propensity score did not meet the criteria for variable selection, and model predictions were similar when adding a propensity score as an additional covariate as a sensitivity analysis (ESM Fig. 5). The variable selection and performance of the propensity score model are reported in ESM (ESM Fig. 6–7).

In the development cohort, the mean CATE across all individuals was a 0.1 mmol/mol (95% credible interval [CrI] −0.3, 0.5) (0.01% [95% CrI −0.03, 0.05]) benefit with GLP1-RA over SGLT2i, suggesting similar average efficacy of both therapies. However, between individuals, there was marked heterogeneity in the predicted CATE estimates (Fig. 1a), with the model predicting a mean HbA_1c_ benefit on SGLT2i therapy for 13,110 (48%) individuals and on GLP1-RA for 14,209 (52%) individuals. In the development cohort, 4787 (17.5%) had a predicted HbA_1c_ benefit >3 mmol/mol (0.3%) (3 mmol/mol is used widely as minimally important difference in clinical trials) with SGLT2i over GLP1-RA, and 5551 (20.3%) had a predicted HbA_1c_ benefit >3 mmol/mol with GLP1-RA over SGLT2i.

### Model calibration

Calibration by decile of model-predicted CATE estimates was good in the development cohort (*n*=27,319; Fig. 1b), the hold-back CPRD validation cohort (*n*=19,075, Fig. 1c), and in propensity-matched cohorts (ESM Fig. 8).

**Fig. 2:**
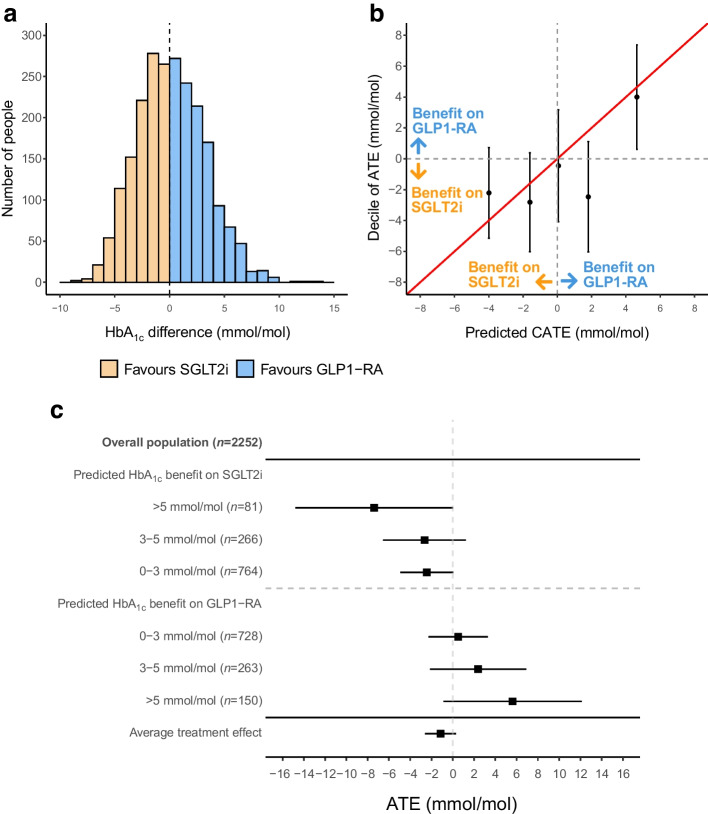
External validation in Tayside & Fife, Scotland (*n*=2252). (**a**) Distribution of CATE estimates for SGLT2i vs GLP1-RA; negative values reflect a predicted glucose-lowering treatment benefit on SGLT2i and positive values reflect a predicted treatment benefit on GLP1-RA. (**b**) Calibration between adjusted ATE and predicted CATE estimates, by quintile of predicted CATE. (**c**) ATE estimates within subgroups defined by clinically meaningful CATE thresholds (SGLT2i benefit >5, 3–5 and 0–3 mmol/mol, GLP1-RA benefit >5, 3–5 and 0–3 mmol/mol). Bars represent 95% CrI

In the external Scottish cohort (Tayside & Fife; *n*=2252 [1837 initiating SGLT2i, 415 initiating GLP1-RA]; baseline characteristics: ESM Table 1), a similar distribution of predicted CATE to CPRD was observed (Fig. 2a), and there was a clear difference between upper (favouring GLP1-RA) and lower (favouring SGLT2i) quintiles, but modest calibration in middle quintiles (Fig. 2b). Among 81 (3.6%) individuals with a model-predicted HbA_1c_ benefit >5 mmol/mol (>0.5%) for SGLT2i over GLP1-RA, there was a 7.4 mmol/mol (95% CrI 0.1, 14.8) (0.7% [95% CrI 0, 1.4]) benefit for SGLT2i (Fig. 2c). In contrast, among 150 (6.7%) individuals with a model-predicted HbA_1c_ benefit >5 mmol/mol for GLP1-RA over SGLT2i, there was a 5.6 mmol/mol (95% CrI −0.9, 12.1) (0.5% [95% CrI −0.1, 1.1]) benefit for GLP1-RA.

### Model interpretability

**Fig. 3:**
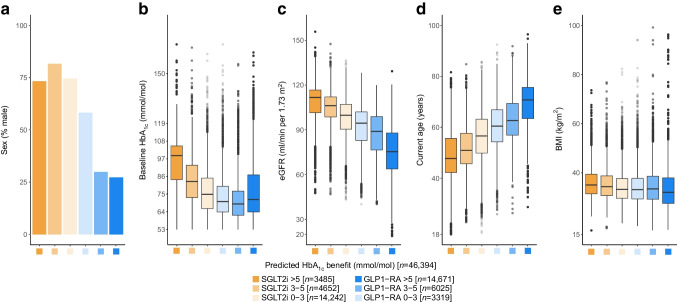
Distributions of major clinical characteristics predicting differential HbA_1c_ outcome with SGLT2i and GLP1-RA. Distributions of key differential clinical characteristics in the combined development and validation cohorts (*n*=46,394 with complete predictor data) for subgroups defined by predicted HbA_1c_ outcome differences: SGLT2i benefit >5 mmol/mol, 3–5 mmol/mol and 0–3 mmol/mol, GLP1-RA benefit >5 mmol/mol, 3–5 mmol/mol and 0–3 mmol/mol. The box and whisker plots include median, first and third quartile, with outliers laying further than 1.5 times the interquartile range. (**a**) Percentage of male individuals in each of the subgroups. (**b**) Baseline HbA_1c_. (**c**) eGFR. (**d**) Current age. (**e**) BMI

Stratifying the combined development and validation cohorts (*n*=46,394 with complete predictor data) into subgroups defined by predicted CATE, there were clear differences in clinical characteristics, with those having a greater predicted HbA_1c_ benefit with GLP1-RA over SGLT2i being predominantly female and older, with lower baseline HbA_1c_, eGFR and BMI (Fig. 3a–e, ESM Table 1). SGLT2i were predicted to have a greater HbA_1c_ benefit over GLP1-RA for 32% of those with baseline HbA_1c_ levels <64 mmol/mol (8%), compared to 67% of those with baseline HbA1c ≥86 mmol/mol (≥10%). An evaluation of relative variable importance identified the number of other current glucose-lowering drugs (a higher number of concurrent therapies favouring SGLT2i as the optimal treatment), sex, current age, and to a lesser extent BMI and HbA_1c_ as the most influential predictors (relative importance ≥3%). In contrast, microvascular complications and cardiovascular comorbidities had very modest effects on differential response (ESM Fig. 9).

### Replication of sex differences in glycaemic response in clinical trials

**Fig. 4:**
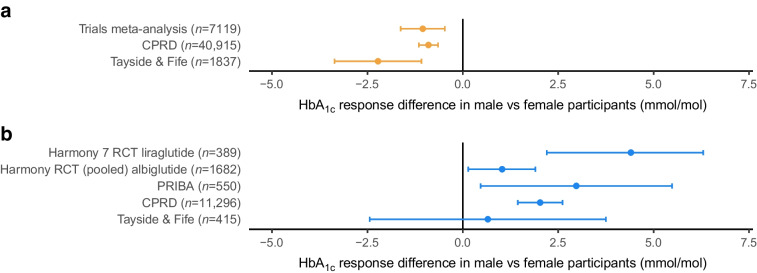
Differences in HbA_1c_ outcome by sex, in randomised clinical trial and observational datasets. All estimates are adjusted for baseline HbA_1c_. Estimates lower than zero represent a greater HbA_1c_ reduction in male compared with female participants. Bars represent 95% CrI. (**a**) SGLT2i: point estimates for the trials meta-analysis and CPRD are reproduced from Dennis et al (2022) [6]. (**b**) GLP1-RA

Whilst previous analyses of clinical trials and observational data for SGLT2i have shown a modestly greater HbA_1c_ response in men compared with women, which we additionally reproduced in Tayside & Fife (Fig. 4a,b), sex differences in GLP1-RA response have not been clearly established. Here, we focused on individual-level randomised clinical trial data of GLP1-RA from the HARMONY programme (liraglutide [*n*=389] and albiglutide [*n*=1682]) [18], the PRIBA prospective cohort study (non-insulin treated participants only: liraglutide [*n*=350], exenatide [*n*=197], lixisenatide [*n*=3]) [14], and Tayside & Fife (*n*=415). Baseline characteristics for the cohorts are reported in ESM Table 1. Across all studies, there was consistent evidence of a greater baseline HbA_1c_ adjusted glycaemic response in women vs men; this was most marked for liraglutide in the HARMONY 7 trial [7] where a 4.4 mmol/mol (95% CrI 2.2, 6.3) (0.4% [95% CrI 0.2, 0.6]) greater response in women vs men was observed.

### Effect of targeting therapy based on differential HbA_1c_ outcome on other short- and long-term outcomes

Specific subpopulations were defined for each short-term outcome to maximise the number of eligible individuals for each analysis and based on the availability of observed outcome data (12 month HbA_1c_ change from baseline [to evaluate absolute response] *n*=87,835; 12 month weight change *n*=41,728; treatment discontinuation within 6 months [a proxy for tolerability] *n*=77,741) (ESM Fig. 2). Longer-term outcomes were evaluated up to 5 years from drug initiation, excluding individuals with a history of cardiovascular disease or CKD for MACE, heart failure, and adverse kidney (composite of ≥40% decline in eGFR or kidney failure [14]) outcomes (*n*=52,052) and individuals with a history of retinopathy, neuropathy and nephropathy for microvascular outcome (*n*=34,524). (ESM Fig. 2).

**Fig. 5:**
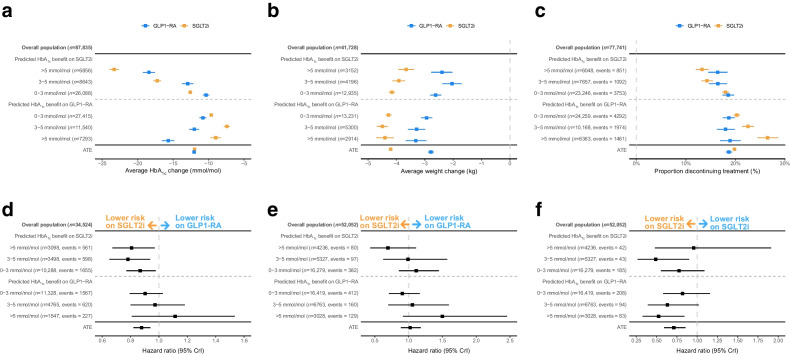
Differences in short-term and long-term clinical outcomes with SGLT2i and GLP1-RA for subgroups defined by predicted HbA_1c_ response differences. (**a**) Twelve month HbA_1c_ change from baseline. (**b**) Twelve month weight change. (**c**) Six month risk of discontinuation. (**d**) HR for 5 year risk of new-onset microvascular complications (retinopathy, nephropathy or neuropathy). (**e**) HR for 5 year relative risk of MACE. (**f**) HR for 5 year risk of heart failure. HRs represent the relative risk for those treated with GLP1-RA in comparison with SGLT2i therapy, with a value under 1 favouring SGLT2i therapy. Data underlying the figure are reported in ESM Table 3. Bars represent 95% CrI

For HbA_1c_ change from baseline, of the 6856 individuals (7.8%) with a predicted HbA_1c_ benefit on SGLT2i of >5 mmol/mol (>0.5%), those who received SGLT2i had a 23.3 mmol/mol (95% CrI 22.6, 24.0) (2.1% [95% CrI 2.1, 2.2]) mean reduction in HbA_1c_ and those who received GLP1-RA had an 18.4 mmol/mol (95% CrI 17.6, 19.3) (1.7% [95% CrI 1.6, 1.8]) mean reduction in HbA_1c_ (Fig. 5a). In contrast, of the 7293 individuals (8.3%) with a predicted HbA_1c_ benefit on GLP1-RA of >5 mmol/mol, those receiving GLP1-RA had a 15.7 mmol/mol (95% CrI 14.8, 16.6) (1.4% [95% CrI 1.4, 1.5]) mean reduction in HbA_1c_, and those receiving SGLT2i had a 9.0 mmol/mol (95% CrI 8.2, 9.7) (0.8% [95% CrI 0.8, 0.9]) mean reduction in HbA_1c_. Consistent differences were observed in individuals of South Asian, Black, Other and Mixed ethnicity (ESM Fig. 10), and those with and without a history of cardiovascular disease (ESM Fig. 11).

Observed weight change was consistently greater for individuals treated with SGLT2i compared with GLP1-RA across all subgroups (Fig. 5b). Short-term discontinuation was lower in those treated with the drugs predicted to have the greatest glycaemic benefit, mainly reflecting differences in SGLT2 discontinuation across predicted levels of differential glycaemic response (Fig. 5c). Relative risk of new-onset microvascular complications also varied by subgroup, with a lower risk with SGLT2i vs GLP1-RA only in subgroups predicted to have a glycaemic benefit with SGLT2i (Fig. 5d). HRs for the risk of new-onset MACE were similar overall (HR 1.02 [95% CrI 0.89, 1.18]) and by subgroup (Fig. 5e). HRs for the risks of both new-onset heart failure and adverse kidney outcomes were lower with SGLT2i (heart failure HR 0.71 [95% CrI 0.59, 0.85]; CKD HR 0.41 [95% CrI 0.30, 0.56]) with no clear evidence of a difference by subgroup (Fig. 5f, ESM Fig. 12). Results for all outcomes were consistent in propensity-matched cohorts (ESM Fig. 13–14).

### Comparison of model predictions with our previously published treatment selection model for SGLT2i and DPP4i therapies

Predictions for HbA_1c_ response with SGLT2i from the SGLT2i v GLP1-RA treatment selection model were highly concordant (*R*^2^ >0.92) with those from our recently published SGLT2i vs DPP4i treatment selection model [6] (ESM Fig. 15). Estimating differential HbA_1c_ responses using both models in our study population with complete data (*n*=82,933) suggested SGLT2i is the predicted optimal therapy for HbA_1c_ in 48.2% (*n*=39,975) of individuals, GLP1-RA the predicted optimal therapy in 51.3% (*n*=42,519), and DPP4i the optimal therapy for only 0.5% (*n*=439).

### Prototype treatment selection model

A prototype treatment selection model web calculator providing individualised predictions of differences in HbA_1c_ outcomes is available at: https://pm-cardoso.shinyapps.io/SGLT2_GLP1_calculator/.

## Discussion

We have developed and validated a novel treatment selection algorithm using state-of-the-art Bayesian methods to predict differences in one-year glycaemic outcomes for SGLT2i and GLP1-RA therapies. Our evaluation shows that glycaemic response-based targeting of these two major drug classes to individuals with type 2 diabetes based on their characteristics can not only optimise glycaemic control, but may also associate with improved tolerability and reduced risk of new-onset microvascular complications. In contrast, we found limited evidence for heterogeneity in other clinical outcomes, with overall equipoise between the two therapies for new-onset MACE and a clear overall benefit with SGLT2i over GLP1-RA for new-onset heart failure and adverse kidney outcomes independent of differences in glycaemic efficacy (differences which themselves reflect differences in the clinical characteristics of individual patients). Predictions are based on routine clinical characteristics, meaning the model could be deployed in many countries worldwide where these agents are available, without the need for additional testing.

Our approach differs from notable recent studies that have attempted to subclassify people with type 2 diabetes or used dimensionality reduction to represent type 2 diabetes heterogeneity [6, 27, 28]. Whilst these approaches can provide important insight into underlying heterogeneity of type 2 diabetes, they, by definition, lose information about the specific characteristics of individual patients, meaning they could be suboptimal for accurately predicting the treatment or disease progression outcomes for individuals [29]. If subclassification approaches based on clinical features are to have potential clinical utility, they will need to be updated over time as an individual’s phenotype evolves [30]. In contrast, our ‘outcomes-based’ approach enables the prediction of optimal therapy when a treatment decision is made, uses the specific information available for a patient at that point in time and avoids subclassification.

Although BCF models are only causal under specific assumptions [31], our study might provide insights into differences in the possible underlying mechanisms of action of GLP1-RA and SGLT2i, and the clinical utility of these differences. The strongest predictor of a differential glycaemic response was the number of currently prescribed glucose-lowering therapies, which is a likely proxy of the degree of diabetes progression (and, therefore, underlying beta cell failure) of an individual. A plausible biological explanation for this proxy is an attenuated GLP1-RA response in individuals with markers of beta cell failure including longer diabetes duration and lower fasting C-peptide, as previously demonstrated in a prospective population-based analysis [7], with no evidence of differences for SGLT2i [31]. Whilst in contrast, post hoc analyses of clinical trials have found type 2 diabetes duration and beta cell function do not modify glycaemic outcomes with GLP1-RA [19, 32, 33], this may reflect trial inclusion criteria as participants had relatively higher beta cell function compared with population-based cohorts [34]. The favouring of GLP1-RA over SGLT2i in women is novel but is supported by our trial validation and recent pharmacokinetic data demonstrating higher circulating GLP1-RA drug concentrations and, consequently, greater HbA_1c_ reduction in female compared with male participants [33]. For SGLT2i, increased urinary glucose excretion likely explains the greater relative glycaemic efficacy with higher baseline HbA_1c_ and eGFR, which, in concordance with our analysis, has been previously demonstrated in trial data [35]. Given the lack of previous studies evaluating whether the relative glucose-lowering efficacy of the two drug classes is altered by baseline HbA_1c_ [6], an interesting finding is that our model suggests a greater relative glycaemic benefit with SGLT2i over GLP1-RA at higher baseline HbA_1c_ levels, which warrants further study. Of note, the comorbidities included in the final model had modest effects on HbA_1c_ and are likely to be proxy measures of factors underlying differential response to these therapies.

A further interesting finding is that mean HbA_1c_ response on both drug classes was similar, and weight loss slightly greater with SGLT2i, in contrast to RCTs where network meta-analysis suggests a greater glycaemic and weight efficacy of most individual GLP1-RA over SGLT2i [12, 36, 37]. The relative average equipoise between the two drug classes in our study is likely indicative of a diminished real-world response to GLP1-RA, a phenomenon also documented in other real-world studies [37, 38], which may relate to reduced real-world adherence to GLP1-RA [38].

Our study represents the second application of our novel validation framework for precision medicine models, which, in the absence of true observed outcomes (for an individual patient on one therapy, the counterfactual outcome they would have had on an alternative therapy cannot be observed [39]), evaluates accuracy in subgroups defined by predicted CATE. The previous study developed a treatment selection model for SGLTi2 vs DPP4i therapy in an independent dataset. Although this previous model demonstrated marked heterogeneity in the relative glycaemic outcome, most (84%) individuals had a greater glycaemic reduction with SGLT2i. In contrast, this GLP1-RA/SGLT2i model shows greater heterogeneity in treatment effects but with equipoise on ATE between the two therapies (52% favouring GLP1-RA). Furthermore, we demonstrate that optimising therapy based on predicted glycaemic response may lower microvascular complication risk, a finding concordant with evidence from the UKPDS study on the importance of good glycaemic control to lower the risk of microvascular disease [23, 40].

Further developments to this model could include the incorporation of non-routine and pharmacogenetic markers (recently identified for GLP1-RA) [41], and additional glucose-lowering drug classes, in particular, off-patent sulfonylureas and pioglitazone, to support the deployment of the algorithm in lower-income countries where the availability of newer medications may be limited. Assessment of semaglutide, a GLP1-RA with potent glycaemic effect excluded here due to low numbers prescribed during the period of data availability, and tirzepatide, a dual glucose-dependent insulinotropic polypeptide (GIP) and GLP-1 receptor agonist not currently available in the UK, is an important area for future research as our model may benefit from recalibration for these newer therapies. Although our ethnicity-specific validation suggests good performance in individuals of South Asian, Black, Other and Mixed ethnicity, setting and ethnicity-specific validation and optimisation would also improve future clinical utility. Given the possibility of selection bias due to non-random treatment assignment, validation in a dataset where individuals were randomised to therapy would further strengthen the evidence for model deployment. However, few active comparator trials of these two drug classes have been conducted [8] and, to our knowledge, none are available for data sharing. Ultimately, research, likely in even larger datasets, is needed on whether individualised models for other short- and long-term outcomes beyond glycaemia, particularly cardiorenal disease, can further improve current prescribing approaches [42]. Finally, a limitation of our study is that despite being state-of-the-art and with a key advantage of allowing estimation of predictions with uncertainty, and so facilitating more transparent evaluation, the BCF methods we applied are subject to ongoing development in several key areas such as variable selection [18, 19], scalability and handling of missing data [20].

In conclusion, our study demonstrates a clear potential for targeted prescribing of GLP1-RA and SGLT2i to individual people with type 2 diabetes based on their clinical characteristics to improve glycaemic outcomes, tolerability and risk of microvascular complications. This provides an important advance on current type 2 diabetes guidelines, which only recommend preferentially prescribing these therapies to individuals with, or at high risk of, cardiorenal disease, with no clear evidence to choose between the two drug classes. Precision type 2 diabetes prescribing based on routinely available characteristics has the potential to lead to more informed and evidence-based decisions on treatment for people with type 2 diabetes worldwide in the near future.

### Supplementary Information

Below is the link to the electronic supplementary material.

Supplementary file1 (PDF 3535 KB)

## Data Availability

The UK routine clinical data analysed during the current study are available in the CPRD repository (CPRD; https://cprd.com/research-applications), but restrictions apply to the availability of these data, which were used under license for the current study, and so are not publicly available. For re-using these data, an application must be made directly to CPRD. Data from Scotland are anonymised real-world medical records available by request through the Scottish Care Information-Diabetes Collaboration, Tayside & Fife, Scotland unit (https://www.sci-diabetes.scot.nhs.uk/). Clinical trial data are not publicly available for access an application must be made directly to GSK and www.ClinicalStudyDataRequest.com.

## Abbreviations

95% CrI: 95% Credible interval
ALT: Alanine aminotransferase
ATE: Average treatment effect
BCF: Bayesian causal forest
CATE: Conditional average treatment effect
CKD: Chronic kidney disease
CPRD: UK Clinical Practice Research Datalink
DPP4i: DPP4 inhibitors
GLP-1: Glucagon-like peptide-1
GLP1-RA: Glucagon-like peptide-1 receptor agonists
MACE: Major adverse cardiovascular events
SGLT2i: Sodium–glucose cotransporter 2 inhibitors

## Code availability

All R code used for the analysis is provided at https://github.com/Exeter-Diabetes/CPRD-Pedro-SGLT2vsGLP1.
