# Supplementary material for: Phenotype-based targeted treatment of SGLT2 inhibitors and GLP-1 receptor agonists in type 2 diabetes
Source: Diabetologia. 2024 Feb 22;67(5):822–36. doi: 10.1007/s00125-024-06099-3 (PMC10955037; doi:10.1007/s00125-024-06099-3)
Supplement: Supplementary file 1 — Supplementary file1 (PDF 3535 KB) [file 125_2024_6099_MOESM1_ESM.pdf]

Supplementary material: Phenotype-based targeted treatment of SGLT2 inhibitors and GLP-1 receptor agonists in type 2 diabetes

Authors: Pedro Cardoso , Katie G. Young, Anand T.N. Nair, Rhian Hopkins, Andrew McGovern, Eram Haider, Piyumanga Karunaratne, Louise Donnelly, Bilal A Mateen, Naveed Sattar, Rury R. Holman, Jack Bowden, Andrew T. Hattersley, Ewan R. Pearson, Angus G. Jones, Beverley M. Shields, Trevelyan J. McKinley, John M. Dennis, on behalf of the MASTERMIND consortium

Table of Contents

ESM Methods 4

Model Overview 4

Sparse BCF 5

Propensity score estimation 7

Variable selection 7

Final model fit 7

Variable importance 8

Model validation: sensitivity analysis using propensity scores 8

Secondary outcomes 8

ESM Results 9

ESM Table 1: Baseline clinical characteristics of patients initiating GLP-1 receptor agonists and SGLT2-inhibitors from the UK Clinical Practice Research Datalink for model development and validation cohorts. 9

a) CPRD HbA_{1c} model development cohort, n= 31,346. 9

b) CPRD HbA_{1c} model validation cohort, n= 20,865. 11

c) Tayside & Fife (Scotland) routine clinical data, n= 2,252. 13

d) Clinical trials and prospective cohorts. 14

e) CPRD patient subgroups defined by predicted HbA_{1c} benefit >5 mmol/mol (>2.6%) with either SGLT2i or GLP-1RA, n= 14,149 (values for Fig. 3) 15

ESM Table 2: Model performance statistics for predicting HbA_{1c} outcome with 95% credible intervals 17

ESM Table 3: Data underlying Fig. 5, showing differential treatment effects for secondary clinical outcomes across subgroups defined by clinical cut-offs of predicted treatment effects. 18

a) Predicted HbA_{1c} change (n=87,835) 18

b) Predicted weight change (n=41,728), with additional adjustment for baseline weight 18

c) Risk of discontinuation (n=77,741) 18

d) Microvascular complications (n=34,524), with additional adjustment for baseline cardiovascular risk 19

e) Major adverse cardiovascular events (MACE) (n=52,052), with additional adjustment for baseline cardiovascular risk 19

f) Heart Failure (n=52,052), with additional adjustment for baseline cardiovascular risk 19

g) Developing chronic kidney disease stage 5 or drop of eGFR by 40% (n=52,052), with additional adjustment for baseline cardiovascular risk 20

ESM Fig. 1: CPRD patient flow and inclusion criteria for the development of the treatment selection model. 21

ESM Fig. 2: CPRD patient flowchart and inclusion criteria for the analysis of additional outcomes. 22

ESM Fig. 3: Variables selected for the prognostic (factors predictive of HbA_{1c} response to SGLT2i therapy) component (A) and the moderator (factors predictive of differential HbA_{1c} response with GLP1-RA compared to SGLT2i therapies) component (B) of the model. 23

ESM Fig. 4: Comparison of predicted outcome HbA_{1c} and predicted conditional average treatment effect (CATE) estimates from a sparse Bayesian Causal Forest (BCF) model and a BCF model with different sets of variables. 24

ESM Fig. 5: Comparison of predicted outcome HbA_{1c} and predicted conditional average treatment effect (CATE) estimates from two Bayesian Causal Forest (BCF) models with and without including propensity scores in the development cohort. 25

ESM Fig. 6: Variable selection plot for the propensity score model — identifying the most important predictors. 26

ESM Fig. 7: Received operating characteristic (ROC) and precision-recall curves of the propensity score model developed in the development cohort and validated in hold-out validation data. 27

ESM Fig. 8: Calibration plots of predicted conditional treatment effect (CATE) estimates using propensity score matching. 28

ESM Fig. 9: Model interpretability plots 29

ESM Fig. 10: Differential HbA_{1c} treatment effects in individuals of white ethnicity and people of colour 30

ESM Fig. 11: Calibration plots of predicted conditional individualised treatment effects in the validation cohort for those with and without cardiovascular disease (CVD). 31

ESM Fig. 12: Relative risk of developing new onset chronic kidney disease (CKD) over 5 years, across subgroups defined by clinical cut-offs of predicted treatment benefit. 32

ESM Fig. 13: Short-term and long-term clinical outcomes, across subgroups defined by clinical cut-offs of predicted treatment benefit, in propensity score matched cohorts. 33

ESM Fig. 14: Short-term and long-term clinical outcomes, across subgroups defined by clinical cut-offs of predicted treatment benefit, in propensity score matched cohort with additional covariate adjustment. 34

ESM Fig. 15: Evaluation of concordance between estimates of predicted HbA_{1c} outcome with SGLT2i from our model and a recently published SGLT2i-DPP4-inhibitor treatment selection model[13]. 35

References 37

PRIBA Study Group 38

MASTERMIND consortium 39

ESM Methods

Model Overview

The model development process consisted of a first step of propensity score estimation and a second step of developing a treatment selection model using a Bayesian additive regression tree (BART) framework [1, 2]. We aimed to balance predictive accuracy with model parsimony and build the model around a limited number of routinely collected variables, thus facilitating its use in clinical practice. We used the *bcf* [3] and *sparseBCF* [2] packages in R to fit the model(s) using Markov chain Monte Carlo (MCMC). By default, *bcf* places stronger regularisation on the moderator side of the model, shrinking the treatment effects towards homogeneity where there is a lack of strong evidence to the contrary. For this model, we are comparing two therapies against each other (rather than a therapy to a control group), so we used the same prior structure for the prognostic and moderator parts of the model.

The Bayesian additive regression trees (BART) technique employs a Bayesian methodology for non-parametric function estimation through regression trees[4]. These trees employ a recursive binary partitioning of the predictor space, creating a series of hyperrectangles to approximate an unknown function f. The predictor space's dimension equals the number of variables, p. Due to their flexible fitting of interactions and non-linearities, regression tree models are helpful. Models composed of multiple regression trees can capture interactions, non-linearities, and additive effects in f.

BART can be considered a sum-of-trees ensemble, with a novel estimation approach relying on a fully Bayesian probability model. Specifically, the BART model can be expressed as:

$$\boldsymbol{Y} = f(\boldsymbol{X}) + \boldsymbol{\varepsilon} \approx \boldsymbol{T}_1^{\boldsymbol{M}}(\boldsymbol{X}) + \boldsymbol{T}_2^{\boldsymbol{M}}(\boldsymbol{X}) + \cdots + T_m^{M}(\boldsymbol{X}) + \boldsymbol{\varepsilon}, \qquad \boldsymbol{\varepsilon} \sim \boldsymbol{N}_n\left(\boldsymbol{0}, \boldsymbol{\sigma}^2 \boldsymbol{I}_n\right) \tag{1}$$

In this context, $\boldsymbol{Y}$ represents the response vector with dimensions $n \times 1$, while $\boldsymbol{X}$ is the $n \times p$ design matrix with column-joined predictors. Additionally, $\boldsymbol{\varepsilon}$ represents the $n \times 1$ noise vector. The analysis involves m separate regression trees, each consisting of a tree structure (referred to as $\mathbf{T}$) and the parameters at the terminal nodes or leaves (referred to as $\mathbf{M}$). When combined, both components make up the complete tree denoted as $\mathbf{T}^{\mathbf{M}}$. This complete representation encompasses both the structure and set of leaf parameters.

In the BART model, the prior consists of three distinct components. Firstly, it includes the tree structure itself. Secondly, it incorporates the leaf parameters given the tree structure. Finally, it encompasses the error variance $\boldsymbol{\sigma}^2$, independent of the tree structure and leaf parameter.

$$\mathbb{P}(\mathbf{T}_1^{\mathbf{M}}, \dots, \mathbf{T}_m^{\mathbf{M}}, \boldsymbol{\sigma}^2) = \left[\prod_t \mathbb{P}\left(\mathbf{T}_t^{\mathbf{M}}\right)\right] \mathbb{P}\left(\boldsymbol{\sigma}^2\right) = \left[\prod_{\boldsymbol{t}} \mathbb{P}(\mathbf{M}_t|\mathbf{T}_t)\mathbb{P}(\mathbf{T}_t)\right] \mathbb{P}(\boldsymbol{\sigma}^2)$$
$$= \left[\prod_t \prod_l \mathbb{P}\left(\boldsymbol{\mu}_{t,l}\middle|\mathbf{T}_t\right)\mathbb{P}(\mathbf{T}_t)\,\mathbb{P}(\boldsymbol{\sigma}^2)\right]$$

Initially, we discuss $\mathbb{P}(\mathbf{T}_t)$, a component of the prior that impacts node placement within the tree. The probability of nonterminal nodes at a particular depth d is $\alpha(1+d)^{-\beta}$ where $\alpha \in (0,1)$ and $\beta \in [0, \infty]$. In this context, depth is measured as the distance from the root. For instance, the root node has a depth of 0, while its first child node has a depth of 1, and so on. This prior form can limit the

complexity of any single tree by enforcing shallow tree structures. The values for these hyperparameters are $\alpha = 0.95$ and $\beta = 2$, in line with Chipman *et al. [4]*.

Next, we discuss the prior component $\mathbb{P}(\mathbf{M}_t|\mathbf{T}_t)$ which regulates the leaf parameters. When a tree has a collection of terminal nodes, each node (or leaf) has a continuous parameter representing the "best guess" of the response in that specific partition of predictor space. This parameter is the fitted value assigned to any observation within that node. The prior on each of the leaf parameters is $\boldsymbol{\mu}_l \sim^{iid} \boldsymbol{N}(\boldsymbol{\mu}_{\boldsymbol{\mu}}/m, \boldsymbol{\sigma}_{\boldsymbol{\mu}}^2)$, where the expectation, $\boldsymbol{\mu}_{\boldsymbol{\mu}}$, is selected as the range centre, specifically $(y_{\min} + y_{\max})/2$. However, outliers can impact the range centre, which can be remedied by logging the response or applying windsorisation.

The variance is chosen empirically such that the range centre plus or minus $k = 2$ variances cover 95% of the response values provided in the training set (where $k = 2$ corresponds to 95% coverage). Therefore, given m trees, we automatically employ $\boldsymbol{\sigma}_{\boldsymbol{\mu}}$ such that $m\boldsymbol{\mu}_{\boldsymbol{\mu}} - k\sqrt{m}\boldsymbol{\sigma}_{\boldsymbol{\mu}} = y_{\min}$ and $m\boldsymbol{\mu}_{\boldsymbol{\mu}} + k\sqrt{m}\boldsymbol{\sigma}_{\boldsymbol{\mu}} = y_{\max}$. This prior aims to achieve model regularisation by shrinking the leaf parameters towards the centre of the response distribution.

The last prior is on the error variance, and it is selected as $\boldsymbol{\sigma}^2 \sim \text{InvGamma}(\nu/2, \nu\lambda/2)$. λ is calculated from the data to ensure that there is a $q = 90\%$ probability that the BART model will outperform the RMSE of an ordinary least squares regression. Consequently, the majority of the prior probability mass is positioned beneath the RMSE from the least squares regression. Furthermore, this prior restricts the likelihood of small $\boldsymbol{\sigma}^2$ values to prevent overfitting.

To generate draws from the posterior distribution of $\mathbb{P}\left(\mathbf{T}_1^{\mathbf{M}}, \dots, \mathbf{T}_m^{\mathbf{M}}, \boldsymbol{\sigma}^2 \middle| \boldsymbol{\mu}\right)$, a Gibbs sampler is utilised. A key feature of the Gibbs sampler for BART is to employ a form of "Bayesian backfitting" where the jth tree is fit iteratively, holding all other $m - 1$ trees constant by exposing only the residual response that remains unfitted. This relies on Metropolis-Hastings draws from the posterior of the tree distributions, which involve introducing minor perturbations to the tree structure, such as growing a terminal node by adding two child nodes, pruning two child notes to render their parent node terminal, or modifying a split rule. These three potential tree adjustments are labelled as GROW, PRUNE, AND CHANGE. The prior probabilities for proposing changes to the tree structures are: GROW $= 2.5/9$, PRUNE $= 2.5/9$ and CHANGE $= 4/9$.

BART was modified to handle classification problems for categorical response variables. For the binary classification problem (coded with outcomes "0" and "1"), we assume a probit model:

$$\mathbb{P}(\boldsymbol{Y} = 1|\boldsymbol{X}) = \boldsymbol{\Phi}\left(\mathbf{T}_1^{\mathbf{M}}(\boldsymbol{X}) + \mathbf{T}_2^{\mathbf{M}}(\boldsymbol{X}) + \cdots + \mathbf{T}_m^{\mathbf{M}}(\boldsymbol{X})\right),$$

where $\boldsymbol{\Phi}$ denotes the cumulative density function of the standard normal distribution. In this formulation, the sum-of-trees model serves as an estimate of the conditional probit at $\boldsymbol{x}$, which can be easily transformed into a conditional probability estimate of $Y = 1$. The prior on $\boldsymbol{\sigma}^2$ is not needed in the classification setting as the model assumes $\boldsymbol{\sigma}^2 = 1$. The prior on the tree structure remains the same as in the regressions setting, and a few minor modifications are required for the prior on the leaf parameters. See Chipman *et al.* (2010)[4] and Kapelner *et al.* (2013)[5] for more details.

Sparse BCF

Bayesian Additive Regression Trees (BART) are a non-parametric regression model used to estimate conditional expectations of a response variables Y_i via a "sum-of-trees". We can redefine equation (1) to be:

$$Y_i = f(\boldsymbol{X}_i, Z_i) + \varepsilon_i, \quad \text{where } \varepsilon_i \sim N(0, \sigma^2)$$

Considering this regression framework, we can use BART to flexibly represent $f(\,.\,)$ as

$$f(\boldsymbol{X}, Z) = \sum_{j=1}^{m} g_j\left([\boldsymbol{X} \ \ Z], (T_j, M_j)\right),$$

where m is the total number of trees in the model, and each tree is defined by a pair (T_j, M_j). T_j represents the binary split rules of the tree, while M_j is the set of terminal nodes in that tree. The function $g_j(\,.\,)$ is specific to each tree and maps the predictors $[\boldsymbol{X} \ \ Z]$ to the set of terminal nodes M_j, following the binary split rules in T_j. The conditional mean function $f(\boldsymbol{x}, z) = \mathbb{E}[Y_i | \boldsymbol{X}_i = \boldsymbol{x}_i, Z_i = z_i]$ is calculated by adding up all the terminal nodes ψ_{ij} assigned to the predictors $[\boldsymbol{X} \ \ Z]$ by the tree functions $g_j(\,.\,)$, that is $\sum_{j=1}^{m} g_j(\,.\,)$ Error! Bookmark not defined..

This BART method uses a two-stage regression approach:

$$Z_i \sim \text{Bernoulli}\left(\pi(\widetilde{\boldsymbol{X}}_i)\right), \pi(\widetilde{\boldsymbol{x}}_i) = \mathbb{P}(Z_i = 1 | \widetilde{\boldsymbol{X}}_i = \widetilde{\boldsymbol{x}}_i) \tag{2}$$

$$Y_i = \mu([\boldsymbol{X}_i \ \ \pi(\widetilde{\boldsymbol{X}}_i)]) + \tau(\boldsymbol{W}_i) Z_i + \varepsilon_i \tag{3}$$

The equation (2) deals with propensity score estimation. Equation (3) estimates the prognostic score $\mu(\,.\,)$, defined as the effect of the covariates $\boldsymbol{X}_i \in \mathrm{X}$ on the outcome Y_i in the absence of treatment $\mu(\boldsymbol{x}_i) = \mathbb{E}[Y_i | \boldsymbol{X}_i = \boldsymbol{x}_i, Z_i = 0]$, and CATE $\tau(\,.\,)$.

BART and BCF can effectively handle sparsity due to the random uniform selection of splitting variables. However, they do not explicitly incorporate heterogeneous sparsity or feature shrinkage, which results in the assumption of equal levels of heterogeneity for all covariates included in the model. Let us define first $\boldsymbol{s} = (s_1, \dots, s_p)$ as the vector of splitting probabilities of each predictor $j \in \{1, \dots, P\}$, where each s_j represents the probability for the jth predictor of being chosen as a splitting variable in one of the decision nodes of a tree. Shrinkage BCF places symmetric Dirichlet priors in the estimation of prognostic $\mu(\,.\,)$ and moderating effects $\tau(\,.\,)$ to induce sparsity. For now, we will only consider the case where $\boldsymbol{W}_i = \boldsymbol{X}_i$, that is, where the same set of covariates is used for the estimation of $\mu(\,.\,)$ and $\tau(\,.\,)$. The priors are:

$$\boldsymbol{s}_\mu \sim \text{Dirichlet}\left(\frac{\alpha_\mu}{P+1}, \dots, \frac{\alpha_\mu}{P+1}\right), \qquad \frac{\alpha_\mu}{\alpha_\mu + \rho_\mu} \sim \text{Beta}(a, b)$$

$$\boldsymbol{s}_\tau \sim \text{Dirichlet}\left(\frac{\alpha_\tau}{P}, \dots, \frac{\alpha_\tau}{P}\right), \qquad \frac{\alpha_\tau}{\alpha_\tau + \rho_\tau} \sim \text{Beta}(a, b)$$

where the Beta's parameters are chosen to be $(a, b) = (0.5, 1)$. The hyperparameter ρ is set equal to $(P + 1)$ in the case of the prognostic score, and it is set to $\frac{P}{2}$ in the case of the moderator effect.

The values for leaf hyperparameters are $\alpha = 0.95$ and $\beta = 2$. The tree structure hyperparameters for the prognostic and moderator effects are similar, corresponding to a sum of 200 trees, leaf hyperparameters $= 0.95$ and $\beta = 2$, $\boldsymbol{\sigma}^{\boldsymbol{2}} = 2 \times \mathrm{SD}(Y_i)$ (standard deviation [SD]). See Caron *et al.* (2021) for more details.

For the treatment selection model, we revert the prior on splitting probabilities to a uniform distribution. Otherwise, our application was as described in the previous sections[6].

Propensity score estimation

The BCF developers[3] recommend including a propensity score variable in the prognostic component of BCF models to help alleviate regularisation-induced confounding due to prescribing by indication[3]. We used a standard BART model[7] for the propensity score, fitted using MCMC and the *bartMachine* package in R[7]. All variables extracted initially in the development cohort were used for initial model fitting. However, a subset of the most predictive variables was selected by applying a threshold defined by the proportion of times each predictor was chosen as a spitting rule divided by the total number of splitting rules appearing in the model[7, 8] (ESM Fig. 6). The propensity score model was then refitted with the selected variables. To assess convergence, we monitored the available parameters according to the guidance provided by Kapelner *et al*. [7] and Gelman-Rubin $\hat{R}$ values[9]. The BART propensity score model converged quickly, so we ran 25,000 iterations with the first 15,000 discarded for burn-in; trace plots are available on request and $\hat{R} <$ 1.02. To assess the performance of the final model, received operating characteristic (ROC) and precision-recall curves were fitted to both the development and validation cohorts (ESM Fig. 7).

Variable selection

Variable selection was deployed to develop a parsimonious final model whilst maintaining predictive accuracy. To do this, we used a two-stage approach, wherein the first stage, we built a sparse BCF model[1] incorporating all candidate predictors. Sparse BCF extends standard BCF by replacing the uniform prior distribution placed over the splitting probabilities of each variable (which means that by default, each variable has the same prior probability of being selected for splitting) with a Dirichlet prior over the splitting probabilities. As the model converges, the posterior distribution for these splitting probabilities induces sparsity by assigning higher weight and, thus, higher variable importance to more predictive covariates. This prior was used in the model's prognostic and moderator parts[1]. To define the final predictor set, we selected only variables with a posterior mean splitting probability greater than 1/number of variables. This was a subjective choice, but one we found was sufficient to minimise the number of final predictors without meaningfully affecting predictive accuracy. We ran the sparse BCF model for 250,000 iterations, discarding the first 200,000 iterations as burn-in and monitoring convergence using trace plots (available on request) and Gelman-Rubin $\hat{R}$ values (where all $\hat{R} < 1.01$).

Final model fit

Following variable selection, we fitted a final model using standard BCF without sparsity-inducing priors (since individual-level predictions are not currently possible from the sparse BCF software). The model used 300,000 iterations, discarding the first 200,000 iterations and thinning the remaining iterations by 4, resulting in 25,000 final posterior samples. The propensity score was not included in the final predictor set as it did not meet our threshold for variable selection. As a sensitivity analysis, we refitted the model including the propensity score in the predictor set and compared predictions across the two models (ESM Fig. 4).

Variable importance

Given the known challenge of extracting variable importance from tree-based models, we implemented a pseudo-variable importance measure defined as the proportion of R^2 associated with each variable for predicting the CATE[10]. This was estimated from a linear regression model using all selected variables for the differential part of the model as predictors (with continuous predictors fitted as 3-knot restricted cubic splines) and the predicted CATE as the outcome[11]. To assess how CATE estimates varied across major routine clinical features, we also summarised the marginal distributions of sex, baseline HbA_{1c}, eGFR, current age, and BMI across subgroups defined by the degree of predicted glycaemic differences (SGLT2i benefit of 0–3, 3–5 or >5 mmol/mol (2.2-2.4, 2.4-2.6 or >2.6%); GLP1-RA benefit of 0–3, 3–5 or >5mmol/mol).

Model validation: sensitivity analysis using propensity scores

We employ two other different approaches to estimate the average treatment effects (ATE) within each subgroup:

Propensity score matching: Individuals receiving each drug class within each subgroup were matched by propensity score (the same propensity score used during HbA_{1c} model development), using a caliper distance of 0.05, no replacement and in decreasing order of propensity score values. After defining this restricted patient subset, unadjusted linear regression models were used to estimate the ATE within each subgroup[12].

Propensity score matching with adjustment: The linear regression models of approach 2 were refitted using a double robust approach by adjusting for the full covariate set used in the HbA_{1c} treatment selection model (Table 2 – main manuscript).

Secondary outcomes

HbA_{1c} and weight outcomes were modelled with Bayesian linear regressions, placing a normal distribution of mean 0 and variance 2 on all parameters. Discontinuation was modelled with a Bayesian logistic regression model, placing a normal distribution of mean 0 and a variance 2 on all parameters.

Long-term outcomes (microvascular complications, major adverse cardiovascular events, and heart failure) were modelled with a Bayesian survival model, placing a normal distribution of mean 0 and variance 2 on all parameters.

All models used 10,000 MCMC iterations, discarding the first 5,000 iterations as burn-in for four chains. Convergence was monitored using trace plots and Gelman-Rubin $\hat{R}$ values. Here all $\hat{R} < 1.04$ and trace plots are available on request.

ESM Results

ESM Table 1: Baseline clinical characteristics of patients initiating GLP-1 receptor agonists and SGLT2-inhibitors from the UK Clinical Practice Research Datalink for model development and validation cohorts.
Data are mean [SD] and number (%). Standardised mean difference (SMD). Atherosclerotic cardiovascular disease – composite of myocardial infarction, stroke, ischemic heart disease, peripheral arterial disease and revascularization. *closest values to treatment start in the previous 6 months.

a) CPRD HbA_{1c} model development cohort, n= 31,346.

	GLP-1 receptor agonists (n=6,736)		SGLT2i (n=24,610)		SMD
		Missing (%)		Missing (%)	
Current age, years	57.8 [10.7]		58.5 [10.4]		0.063
Duration of diabetes, years	9.3 [6.4]		9.3 [6.3]		0.002
Year of drug start	2016 [2]		2017 [2]		0.389
Sex					0.149
Male	3,686 (54.7)		15,264 (62.0)		
Female	3,050 (47.3)		9,346 (38.0)		
Ethnicity					0.276
White	5,862 (87.0)		18,914 (76.9)		
South Asian	461 (6.8)		3,480 (14.1)		
Black	202 (3.0)		1,042 (4.2)		
Other	55 (0.8)		376 (1.5)		
Mixed	49 (0.7)		254 (1.0)		
Missing	107 (1.6)		544 (2.2)		
SGLT2i type					
Canagliflozin			4,424 (18.0)		
Dapagliflozin			10,658 (43.3)		
Empagliflozin			9,520 (38.7)		
Ertugliflozin			16 (0.1)		
GLP1-RA type					
Dulaglutide	2,392 (35.5)				
Exenatide (short-acting)	341 (5.1)				
Exenatide (long-acting)	580 (8.6)				
Liraglutide	2,724 (40.4)				
Lixisenatide	703 (10.4)				
Index of multiple deprivation		2 (<0.1)		10 (<0.1)	0.059
1 (Least deprived)	1,099 (16.3)		4,207 (17.1)		
2	1,129 (16.8)		4,423 (18.0)		
3	1,345 (20.0)		4,726 (19.2)		
4	1,433 (21.3)		5,430 (22.1)		
5 (Most deprived)	1,728 (25.7)		5,814 (23.6)		
Smoking status					0.061
Active	1,108 (16.4)		3,977 (16.2)		
Ex-smoker	3,762 (55.8)		13,400 (54.4)		
Non-smoker	1,550 (23.0)		6,240 (25.4)		
Missing	316 (4.7)		993 (4.0)		
Number of glucose-lowering drug classes ever prescribed					0.373
2	823 (12.2)		5,815 (23.6)		
3	1,788 (26.5)		7,734 (31.4)		
4	2,499 (37.1)		4,151 (29.1)		
≥5	1,626 (24.1)		3,910 (15.9)		
Number of other current glucose- -lowering drugs					0.086

0	278 (4.1)		789 (3.2)		
1	2,490 (37.0)		9,898 (40.2)		
2	3,223 (47.8)		11,173 (45.4)		
3	720 (10.7)		2,700 (11.0)		
≥4	25 (0.4)		50 (0.2)		
Background therapy					
Metformin	6,006 (89.2)		22,465 (91.3)		0.071
Sulfonylurea	3,273 (48.6)		9,022 (36.7)		0.243
DPP4i	743 (11.0)		7,184 (29.2)		0.465
SGLT2i	857 (12.7)				
Thiazolidinedione	317 (4.7)		616 (2.5)		0.118
GLP1-RA			1,257 (5.1)		
Biomarkers					
HbA$_{1c}$, mmol/mol*	79.2 [15.6]		76.7 [15.6]		0.161
HbA$_{1c}$, *	9.4 [3.6]		9.2 [3.6]		0.161
BMI, kg/m^2	37.7 [7.0]	122 (1.8)	33.6 [6.7]	781 (3.2)	0.589
eGFR, ml/min per 1.73 m^2	92.6 [18.7]	6 (0.1)	94.9 [14.8]	19 (0.1)	0.139
HDL-cholesterol, mmol/l	1.1 [0.3]	328 (4.9)	1.1 [0.3]	774 (3.1)	0.084
ALT, IU/l	35.8 [20.4]	418 (6.2)	35.2 [20.2]	1,395 (5.7)	0.033
Albumin, g/L	41.6 [3.9]	328 (4.9)	42.1 [3.9]	1,010 (4.1)	0.124
Bilirubin, µmol/l	9.1 [4.7]	265 (3.9)	9.5 [4.9]	914 (3.7)	0.087
Total cholesterol, mmol/l	4.3 [1.1]	17 (0.3)	4.2 [1.1]	28 (0.1)	0.071
Mean arterial BP, mmHg	96.1 [8.8]	12 (0.2)	96.0 [8.8]	36 (0.1)	0.010
Microvascular complications					
Nephropathy	173 (2.6)		461 (1.9)		0.047
Neuropathy	1,828 (27.1)		5,825 (23.7)		0.080
Retinopathy	2,438 (36.2)		9,332 (37.9)		0.036
Cardiovascular conditions					
Angina	768 (11.4)		2,308 (9.4)		0.066
Atherosclerotic CVD	1,420 (21.1)		4,551 (18.5)		0.065
Atrial fibrillation	407 (6.0)		1,094 (4.4)		0.072
Cardiac revascularisation	420 (6.2)		1,520 (6.2)		0.002
Heart failure	380 (5.6)		913 (3.7)		0.092
Hypertension	4,057 (60.2)		13,778 (56.0)		0.086
Ischaemic heart disease	972 (14.4)		3,125 (12.7)		0.051
Myocardial infarction	466 (6.9)		1,546 (6.3)		0.026
Peripheral arterial disease	347 (5.2)		1,041 (4.2)		0.044
Stroke	280 (4.2)		914 (3.7)		0.023
Transient ischaemic attach	152 (2.3)		557 (2.3)		<0.001
Other conditions					
Chronic kidney disease	561 (8.3)		731 (3.0)		0.234
Chronic liver disease	907 (13.5)		2,922 (11.9)		0.048
QRISK2 10-year score	23.5 [13.4]	308 (4.6)	23.2 [12.8]	1,611 (6.5)	0.021
HbA$_{1c}$ outcome					
HbA$_{1c}$, mmol/mol	67.0 [18.2]		64.5 [15.0]		0.152
HbA$_{1c}$, %	8.3 [3.8]		8.1 [3.5]		0.152
Month of HbA$_{1c}$ measure	9.0 [3.5]		9.2 [3.5]		0.067

b) CPRD HbA_{1c} model validation cohort, n= 20,865.

	**GLP-1 receptor agonists (n=6,736)**		**SGLT2i (n=24,610)**		**SMD**
		Missing (%)		Missing (%)	
Current age, years	58.2 [10.8]		58.3 [10.3]		0.009
Duration of diabetes, years	9.3 [6.1]		9.2 [6.3]		0.025
Year of drug start	2016 [2]		2017 [1]		0.378
Sex					0.188
Male	2,460 (53.9)		10,298 (63.2)		
Female	2,100 (46.1)		6,007 (36.8)		
Ethnicity					0.289
White	3.995 (87.6)		12,596 (77.3)		
South Asian	293 (6.4)		2,267 (13.9)		
Black	133 (2.9)		653 (4.0)		
Other	38 (0.8)		249 (1.5)		
Mixed	41 (0.9)		151 (0.9)		
Missing	60 (1.3)		389 (2.4)		
SGLT2i type					
Canagliflozin			2,921 (17.9)		
Dapagliflozin			7,182 (44.0)		
Empagliflozin			6,190 (38.0)		
Ertugliflozin			13 (0.1)		
GLP1-RA type					
Dulaglutide	1,621 (35.5)				
Exenatide (short-acting)	228 (5.0)				
Exenatide (long-acting)	380 (8.3)				
Liraglutide	1,884 (41.3)				
Lixisenatide	449 (9.8)				
Index of multiple deprivation		3 (0.1)		8 (<0.1)	0.043
1 (Least deprived)	784 (17.2)		2,810 (17.2)		
2	823 (18.0)		2,905 (17.8)		
3	878 (19.3)		3,197 (19.6)		
4	949 (20.8)		3,609 (22.1)		
5 (Most deprived)	1,123 (24.6)		3,776 (23.2)		
Smoking status					0.055
Active	760 (16.7)		2,595 (15.9)		
Ex-smoker	2,539 (55.7)		8,890 (54.5)		
Non-smoker	1,057 (23.2)		4,150 (25.5)		
Missing	204 (4.5)		670 (4.1)		
Number of glucose-lowering drug classes ever prescribed					0.378
2	564 (12.4)		3,920 (24.0)		
3	1,232 (27.0)		5,164 (31.7)		
4	1,652 (36.2)		4,663 (28.6)		
≥5	1,112 (24.4)		2,558 (15.7)		
Number of other current glucose- -lowering drugs					0.098
0	199 (4.4)		561 (3.4)		
1	1,682 (36.9)		6,644 (40.7)		
2	2,141 (47.0)		7,364 (45.2)		
3	512 (11.2)		1,692 (10.4)		
≥4	26 (0.6)		44 (0.3)		
Background therapy					
Metformin	4,048 (88.8)		14,961 (91.8)		0.101
Sulfonylurea	2,206 (48.4)		5,851 (35.9)		0.255
DPP4i	505 (11.1)		4,584 (28.1)		0.440
SGLT2i	651 (14.3)				
Thiazolidinedione	195 (4.3)		418 (2.6)		0.094
GLP1-RA			810 (2.6)		

Biomarkers					
HbA_{1c}, mmol/mol*	78.8 [15.5]		76.8 [15.3]		0.138
HbA_{1c}, %*	9.4 [3.6]		9.2 [3.5]		0.138
BMI, kg/m^2	37.4 [6.9]	104 (2.3)	33.7 [6.6]	508 (3.1)	0.549
eGFR, ml/min per 1.73 m^2	91.8 [19.5]	1 (<0.1)	95.2 [14.7]	14 (0.1)	0.196
HDL-cholesterol, mmol/l	1.1 [0.3]	192 (4.2)	1.1 [0.3]	560 (3.4)	0.074
ALT, IU/l	35.9 [21.0]	283 (6.2)	35.5 [20.5]	924 (5.7)	0.018
Albumin, g/L	41.7 [3.9]	197 (4.3)	42.1 [3.9]	725 (4.4)	0.111
Bilirubin, µmol/l	9.1 [4.4]	168 (3.7)	9.6 [5.0]	640 (3.9)	0.101
Total cholesterol, mmol/l	4.3 [1.1]	9 (0.2)	4.2 [1.1]	25 (0.2)	0.087
Mean arterial BP, mmHg	96.1 [9.0]	5 (0.1)	96.2 [8.7]	37 (0.2)	0.011
Microvascular complications					
Nephropathy	112 (2.5)		328 (2.0)		0.030
Neuropathy	1,256 (27.5)		3,780 (23.2)		0.100
Retinopathy	1,706 (37.4)		6,097 (37.4)		<0.001
Cardiovascular conditions					
Angina	540 (11.8)		1,446 (8.9)		0.098
Atherosclerotic CVD	1,086 (23.8)		2,979 (18.3)		0.136
Atrial fibrillation	286 (6.3)		726 (4.5)		0.081
Cardiac revascularisation	319 (7.0)		1,054 (6.5)		0.021
Heart failure	249 (5.5)		600 (3.7)		0.085
Hypertension	2,774 (60.8)		9,089 (55.7)		0.103
Ischaemic heart disease	703 (15.4)		1,979 (12.1)		0.095
Myocardial infarction	319 (7.0)		998 (6.1)		0.035
Peripheral arterial disease	291 (6.4)		675 (4.1)		0.101
Stroke	238 (5.2)		617 (3.8)		0.069
Transient ischaemic attach	148 (3.2)		377 (2.3)		0.057
Other conditions					
Chronic kidney disease	460 (10.1)		447 (2.7)		0.303
Chronic liver disease	603 (13.2)		1,917 (11.8)		0.044
QRISK2 10-year score	24.0 [13.3]	224 (4.9)	23.1 [12.8]	1,056 (6.5)	0.067
HbA_{1c} outcome					
HbA_{1c}, mmol/mol	67.0 [17.8]		64.4 [14.7]		0.161
HbA_{1c}, %	8.3 [3.8]		8.0 [3.5]		0.161
Month of HbA_{1c} measure	8.9 [3.5]		9.2 [3.5]		0.089

c) Tayside & Fife (Scotland) routine clinical data, n= 2,252.

	**GLP-1 receptor agonists (n=415)**		**SGLT2i (n=1,837)**	
		Missing (%)		Missing (%)
Current age, years	58.7 [9.1]		61.5 [9.7]	
Duration of diabetes, years	7.5 [4.2]		7.4 [4.6]	
Year of drug start	2013 [3]		2017 [1]	
Sex				
Male	226 (54.5)		1,155 (62.9)	
Female	189 (45.5)		682 (37.1)	
Ethnicity				
White	415 (100)		1,837 (100)	
Index of multiple deprivation		14 (3.4)		63 (3.4)
1 (Least deprived)	93 (23.2)		375 (21.1)	
2	91 (22.7)		407 (22.9)	
3	74 (18.5)		355 (20.0)	
4	73 (18.2)		348 (19.6)	
5 (Most deprived)	70 (17.5)		289 (16.3)	
Smoking status				
Active	296 (71.3)		1,258 (68.5)	
Non-smoker	119 (28.7)		579 (31.5)	
Number of glucose-lowering drug classes ever prescribed				
2	43 (10.4)		580 (31.6)	
3	125 (30.1)		612 (33.3)	
4	148 (35.7)		412 (22.4)	
≥5	99 (23.9)		233 (12.7)	
Number of other current glucose--lowering drugs				
0 / 1	109 (26.3)		861 (46.9)	
≥2	306 (73.7)		976 (53.1)	
Biomarkers				
HbA_{1c}, mmol/mol	82.8 [16.8]		76.9 [14.3]	
HbA_{1c}, %	9.7 [3.7]		9.2 [3.5]	
BMI, kg/m^2	38.8 [7.4]		34.4 [6.6]	
eGFR, ml/min per 1.73 m^2	93.2 [19.1]		92.5 [16.0]	
HDL-cholesterol, mmol/l	1.1 [0.3]		1.1 [0.3]	
ALT, IU/l	38.1 [21.3]		39.3 [23.2]	
Albumin, g/L	40.9 [4.1]		39.6 [3.8]	
Bilirubin, µmol/l	8.7 [4.1]		9.7 [4.6]	
Total cholesterol, mmol/l	4.4 [1.2]	1 (0.2)	4.3 [1.1]	3 (0.2)
Mean arterial BP, mmHg	100.8 [11.6]	3 (0.7)	97.6 [9.7]	8 (0.4)
Microvascular complications				
Retinopathy	193 (46.5)		779 (42.4)	
Cardiovascular conditions				
Atrial fibrillation	23 (5.5)		85 (4.6)	
Heart failure	19 (4.6)		41 (2.2)	
Myocardial infarction	24 (5.8)		141 (7.7)	
Peripheral arterial disease	2 (0.5)		11 (0.6)	
Stroke	7 (1.7)		40 (2.2)	
Transient ischaemic attach	2 (0.5)		13 (0.7)	
HbA_{1c} outcome				
HbA_{1c}, mmol/mol	68.2 (17.7]		63.5 [13.8]	
HbA_{1c}, %	8.4 [3.8]		8.0 [3.4]	
Month of HbA_{1c} measure	9.5 [3.2]		9.6 [3.3]	

d) Clinical trials and prospective cohorts.

	HARMONY 7 RCT: Liraglutide (n= 389)	HARMONY 7 RCT: Albiglutide (n= 1,682)	PRIBA (n= 550)
Current age, years	55.8 [10.0]	56.4 [10.0]	55.8 [10.3]
Duration of diabetes, years	8.3 [5.6]	8.1 [6.4]	10.0 [6.5]
Sex			
Male	203 (52.2)	873 (52.0)	299 (54.4)
Female	186 (47.8)	809 (48.0)	251 (45.6)
Number of other current glucose- -lowering drugs			
0 / 1	341 (87.6)	1,476 (87.7)	257 (54.4)
≥2	48 (12.4)	206 (12.2)	251 (45.6)
Biomarkers			
HbA_{1c}, mmol/mol	66.0 [14.3]	65.9 [9.9]	82.6 [17.6]
HbA_{1c}, %	8.2 [3.5]	8.2 [3.1]	9.7 [3.8]
BMI, kg/m^2	32.0 [5.9]	32.7 [5.7]	39.6 [26.6]
eGFR, ml/min per 1.73 m^2	95.3 [16.6]	86.5 [20.4]	92.4 [26.6]
HDL-cholesterol, mmol/l	1.2 [0.3]	1.2 [0.3]	1.1 [0.6]
ALT, IU/l	27.7 [13.6]	26.4 [15.7]	34.4 [19.3]
Albumin, g/L	25.9 [37.2]	28.8 [37.5]	41.6 [14.8]
Bilirubin, µmol/l	9.6 [4.3]	9.5 [4.2]	9.5 [6.4]
Total cholesterol, mmol/l	4.5 [1.0]	4.7 [1.1]	4.4 [1.2]
HbA_{1c} outcome			
HbA_{1c}, mmol/mol	54.0 [12.8]	55.0 [13.9]	68.0 [16.8]
HbA_{1c}, %	7.1 [3.3]	7.2 [3.4]	8.4 [3.7]

e) CPRD patient subgroups defined by predicted HbA_{1c} benefit >5 mmol/mol (>2.6%) with either SGLT2i or GLP-1RA, n= 14,149 (values for Fig. 3).

	Predicted benefit on GLP1-RA >5 mmol/mol (>2.6%) (n= 3,319)		Predicted benefit on SGLT2i >5 mmol/mol (>2.6%) (n= 3,485)		SMD
		Missing (%)			
Current age, years	69.4 [9.2]		48.8 [10.1]		2.129
Duration of diabetes, years	11.5 [7.4]		8.1 [5.4]		0.525
Year of drug start	2017 [2]		2017 [2]		0.286
Therapy Taken					0.064
GLP1-RA	956 (28.8)		904 (25.9)		
SGLT2i	2,363 (71.2)		2,581 (74.1)		
Sex					1.033
Male	906 (27.3)		2,550 (73.2)		
Female	2,413 (72.7)		935 (26.8)		
Ethnicity					0.249
White	2,799 (84.3)		2,636 (75.6)		
South Asian	305 (9.2)		555 (15.9)		
Black	133 (4.0)		127 (3.6)		
Other	28 (0.8)		56 (1.6)		
Mixed	21 (0.6)		37 (1.1)		
Missing	33 (1.0)		74 (2.1)		
SGLT2i type					
Canagliflozin	490 (14.8)		453 (13.0)		
Dapagliflozin	845 (25.5)		1,217 (34.9)		
Empagliflozin	1,029 (30.8)		912 (26.2)		
Ertugliflozin	0 (0)		0 (0)		
GLP1-RA type					
Dulaglutide	389 (11.7)		285 (8.2)		
Exenatide (short-acting)	35 (1.1)		57 (1.6)		
Exenatide (long-acting)	71 (2.1)		81 (2.3)		
Liraglutide	384 (11.6)		388 (11.1)		
Lixisenatide	77 (2.3)		92 (2.7)		
Index of multiple deprivation		2 (0.1)		1 (<0.1)	0.249
1 (Least deprived)	671 (20.2)		473 (13.6)		
2	608 (18.3)		525 (15.1)		
3	668 (20.1)		665 (19.1)		
4	680 (20.5)		841 (24.1)		
5 (Most deprived)	690 (20.8)		980 (28.1)		
Smoking status					0.348
Active	346 (10.4)		779 (22.4)		
Ex-smoker	2,019 (60.8)		1,677 (48.1)		
Non-smoker	807 (24.3)		889 (25.5)		
Missing	147 (4.4)		140 (4.0)		
Number of glucose-lowering drug classes ever prescribed					0.450
2	792 (23.9)		276 (7.9)		
3	957 (28.8)		1,144 (32.8)		
4	966 (29.1)		1,307 (37.5)		
≥5	604 (18.2)		758 (21.8)		
Number of other current glucose--lowering drugs					1.477
0	385 (11.6)		25 (0.7)		
1	2,027 (61.1)		467 (13.4)		
2	731 (22.0)		2,346 (67.3)		
3	175 (5.3)		623 (17.9)		
≥4	1 (<0.1)		24 (0.7)		
Background therapy					

Metformin	2,424 (73.0)		3,362 (96.5)		0.690
Sulfonylurea	910 (27.4)		2,046 (58.7)		0.666
DPP4i	556 (16.8)		1,133 (32.5)		0.372
SGLT2i	2,393 (72.1)		2,731 (78.4)		0.146
Thiazolidinedione	57 (1.7)		142 (4.1)		0.141
GLP1-RA	997 (30.0)		1,195 (34.3)		0.091
Biomarkers					
HbA_{1c}, mmol/mol*	78.6 [20.6]		94.5 [15.5]		0.871
HbA_{1c}, %*	9.3 [4.0]		10.8 [3.6]		0.871
BMI, kg/m^2	33.6 [8.9]		36.0 [6.2]		0.310
eGFR, ml/min per 1.73 m^2	74.9 [17.9]		108.4 [13.6]		2.106
HDL-cholesterol, mmol/l	1.2 [0.3]	36 (1.1)	1.0 [0.3]	52 (1.5)	0.661
ALT, IU/l	27.4 [15.4]		40.8 [23.6]		0.672
Albumin, g/L	40.9 [4.0]	23 (0.7)	42.0 [3.9]	22 (0.6)	0.285
Bilirubin, µmol/l	8.8 [4.5]	8 (0.2)	9.6 [5.1]	13 (0.4)	0.179
Total cholesterol, mmol/l	4.3 [1.1]	1 (<0.1)	4.4 [1.1]	1 (<0.1)	0.089
Mean arterial BP, mmHg	94.3 [8.9]		97.1 [8.9]	4 (0.1)	0.315
Microvascular complications					
Nephropathy	90 (2.7)		90 (2.6)		0.008
Neuropathy	1,536 (46.3)		531 (15.2)		0.714
Retinopathy	1,837 (55.3)		1,004 (28.8)		0.558
Cardiovascular conditions					
Angina	472 (14.2)		226 (6.5)		0.256
Atherosclerotic CVD	974 (29.3)		442 (12.7)		0.418
Atrial fibrillation	341 (10.3)		119 (3.4)		0.274
Cardiac revascularisation	242 (7.3)		128 (3.7)		0.159
Heart failure	293 (8.8)		147 (4.2)		0.188
Hypertension	2,317 (69.8)		1,601 (45.9)		0.498
Ischaemic heart disease	580 (17.5)		297 (8.5)		0.269
Myocardial infarction	256 (7.7)		146 (4.2)		0.149
Peripheral arterial disease	332 (10.0)		95 (2.7)		0.301
Stroke	254 (7.7)		103 (3.0)		0.211
Transient ischaemic attach	175 (5.3)		49 (1.4)		0.216
Other conditions					
Chronic kidney disease	832 (25.1)		33 (0.9)		0.768
Chronic liver disease	394 (11.9)		493 (14.1)		0.068
QRISK2 10-year score	32.0 [14.5]	196 (5.9)	18.3 [11.8]	156 (4.5)	1.040
HbA_{1c} outcome					
HbA_{1c}, mmol/mol	67.3 [18.9]		73.5 [18.6]		0.326
HbA_{1c}, %	8.3 [3.9]		8.9 [3.9]		0.326
Month of HbA_{1c} measure	8.7 [3.6]		9.2 [3.5]		0.131

ESM Table 2: Model performance statistics for predicting HbA_{1c} outcome with 95% credible intervals. R^2 and root mean square error (RMSE) were derived from 25,000 iterations post-convergence of the Hba_{1c} treatment selection model.

	Internal validation (development data: n=27,319)	Internal validation (hold-back data: n=19,075)
R^2	0.28 (0.28, 0.29)	0.26 (0.26, 0.27)
RMSE (mmol/mol)	13.4 (13.3, 13.4)	13.4 (13.3, 13.5)

ESM Table 3: Data underlying Fig. 5, showing differential treatment effects for secondary clinical outcomes across subgroups defined by clinical cut-offs of predicted treatment effects.
Estimated values are adjusted for the clinical features used in the treatment selection model (to improve precision and control for potential differences in covariate balance within subgroups).

a) Predicted HbA_{1c} change (n=87,835)

Predicted HbA_{1c} benefit	Predicted HbA_{1c} change (mmol/mol)		
	N patients	Predicted HbA_{1c} change on SGLT2i (95% CI)	Predicted HbA_{1c} change on GLP1-RA (95% CI)
Overall	87,835	-12.0 (-12.1, -11.9)	-12.0 (-12.3, -11.8)
Subgroup			
SGLT2i benefit by >5 mmol/mol	6,856	-23.3 (-24.0, -22.6)	-18.4 (-19.3, -17.6)
SGLT2i benefit by 3-5 mmol/mol	8,643	-17.2 (-17.8, -16.7)	-12.9 (-13.7, -12.2)
SGLT2i benefit by 0-3 mmol/mol	26,088	-12.6 (-12.9, -12.3)	-10.3 (-10.8, -9.9)
GLP1-RA benefit by 0-3 mmol/mol	27,415	-9.6 (-9.9, -9.4)	-10.8 (-11.3, -10.3)
GLP1-RA benefit by 3-5 mmol/mol	11,540	-7.4 (-7.8, -7.0)	-12.0 (-12.7, -11.3)
GLP1-RA benefit by >5 mmol/mol	7,293	-9.0 (-9.7, -8.2)	-15.7 (-16.6, -14.8)

b) Predicted weight change (n=41,728), with additional adjustment for baseline weight

Predicted HbA_{1c} benefit	Predicted weight change (mmol/mol)		
	N patients	Predicted weight change on SGLT2i (95% CI)	Predicted weight change on GLP1-RA (95% CI)
Overall	41,728	-4.20 (-4.3, -4.2)	-2.8 (-2.9, -2.7)
Subgroup			
SGLT2i benefit by >5 mmol/mol	3,152	-3.7 (-3.9, -3.4)	-2.4 (-2.8, -2.0)
SGLT2i benefit by 3-5 mmol/mol	4,196	-3.9 (-4.1, -3.7)	-2.0 (-2.4, -1.7)
SGLT2i benefit by 0-3 mmol/mol	12,935	-4.2 (-4.3, -4.1)	-2.6 (-2.8, -2.4)
GLP1-RA benefit by 0-3 mmol/mol	13,231	-4.3 (-4.4, -4.2)	-2.9 (-3.1, -2.7)
GLP1-RA benefit by 3-5 mmol/mol	5,300	-4.5 (-4.7, -4.3)	-3.3 (-3.6, -3.0)
GLP1-RA benefit by >5 mmol/mol	2,914	-4.4 (-4.7, -4.1)	-3.3 (-3.7, -3.0)

c) Risk of discontinuation (n=77,741)

Predicted HbA_{1c} benefit	Risk of discontinuation (%)		
	N patients	Risk of discontinuation on SGLT2i (95% CI)	Risk of discontinuation on GLP1-RA (95% CI)
Overall	77,741	19.8 (19.5, 20.1)	18.7 (18.1, 19.3)
Subgroup			
SGLT2i benefit by >5 mmol/mol	6,048	13.3 (12.0, 14.6)	16.4 (14.5, 18.4)
SGLT2i benefit by 3-5 mmol/mol	7,657	14.3 (13.1, 15.4)	16.5 (14.7, 18.3)
SGLT2i benefit by 0-3 mmol/mol	23,246	18.0 (17.3, 18.7)	18.6 (17.4, 19.7)
GLP1-RA benefit by 0-3 mmol/mol	24,259	20.3 (19.6, 21.0)	18.7 (17.4, 19.9)
GLP1-RA benefit by 3-5 mmol/mol	10,168	22.5 (21.4, 23.7)	18.0 (16.3, 19.9)
GLP1-RA benefit by >5 mmol/mol	6,363	26.5 (24.4, 28.7)	18.9 (16.9, 21.1)

d) Microvascular complications (n=34,524), with additional adjustment for baseline cardiovascular risk

Predicted HbA_{1c} benefit	Risk of developing microvascular complications - hazard ratio (less than 1 favours SGLT2i) (95% CI)		
	N patients	Events	Treatment difference
Overall	34,524	5,228	0.88 (0.82, 0.94)
Subgroup			
SGLT2i benefit by >5 mmol/mol	3,098	561	0.81 (0.67, 0.97)
SGLT2i benefit by 3-5 mmol/mol	3,498	598	0.78 (0.65, 0.94)
SGLT2i benefit by 0-3 mmol/mol	10,288	1,655	0.87 (0.77, 0.98)
GLP1-RA benefit by 0-3 mmol/mol	11,328	1,567	0.90 (0.79, 1.03)
GLP1-RA benefit by 3-5 mmol/mol	4,765	620	0.97 (0.80, 1.18)
GLP1-RA benefit by >5 mmol/mol	1,547	225	1.11 (0.81, 1.53)

e) Major adverse cardiovascular events (MACE) (n=52,052), with additional adjustment for baseline cardiovascular risk

Predicted HbA_{1c} benefit	Risk of developing MACE - hazard ratio (less than 1 favours SGLT2i) (95% CI)		
	N patients	Events	Treatment difference
Overall	52,052	1,260	1.02 (0.89, 1.18)
Subgroup			
SGLT2i benefit by >5 mmol/mol	4,236	80	0.69 (0.43, 1.11)
SGLT2i benefit by 3-5 mmol/mol	5,327	97	0.99 (0.63, 1.57)
SGLT2i benefit by 0-3 mmol/mol	16,279	382	1.11 (0.86, 1.45)
GLP1-RA benefit by 0-3 mmol/mol	16,419	412	0.91 (0.71, 1.17)
GLP1-RA benefit by 3-5 mmol/mol	6,763	160	1.06 (0.70, 1.60)
GLP1-RA benefit by >5 mmol/mol	3,028	129	1.50 (0.92, 2.46)

f) Heart Failure (n=52,052), with additional adjustment for baseline cardiovascular risk

Predicted HbA_{1c} benefit	Risk of heart failure - hazard ratio (less than 1 favours SGLT2i) (95% CI)		
	N patients	Events	Treatment difference
Overall	52,052	655	0.71 (0.59, 0.85)
Subgroup			
SGLT2i benefit by >5 mmol/mol	4,236	42	0.96 (0.48, 1.91)
SGLT2i benefit by 3-5 mmol/mol	5,327	43	0.49 (0.26, 0.90)
SGLT2i benefit by 0-3 mmol/mol	16,279	185	0.78 (0.55, 1.09)
GLP1-RA benefit by 0-3 mmol/mol	16,419	208	0.82 (0.58, 1.16)
GLP1-RA benefit by 3-5 mmol/mol	6,763	94	0.63 (0.39, 1.02)
GLP1-RA benefit by >5 mmol/mol	3,028	83	0.52 (0.33, 0.84)

g) Developing chronic kidney disease stage 5 or drop of eGFR by 40% (n=52,052), with additional adjustment for baseline cardiovascular risk

Predicted HbA_{1c} benefit	Risk of chronic kidney disease - hazard ratio (less than 1 favours SGLT2i) (95% CI)		
	N patients	Events	Treatment difference
Overall	52,052	185	0.41 (0.30, 0.56)
Subgroup			
SGLT2i benefit by >5 mmol/mol	4,236	13	0.60 (0.20, 1.83)
SGLT2i benefit by 3-5 mmol/mol	5,327	13	0.21 (0.07, 0.64)
SGLT2i benefit by 0-3 mmol/mol	16,279	42	0.30 (0.16, 0.56)
GLP1-RA benefit by 0-3 mmol/mol	16,419	53	0.65 (0.34, 1.22)
GLP1-RA benefit by 3-5 mmol/mol	6,763	36	0.31 (0.16, 0.61)
GLP1-RA benefit by >5 mmol/mol	3,028	28	0.50 (0.22, 1.15)

ESM Fig. 1: CPRD patient flow and inclusion criteria for the development of the treatment selection model.

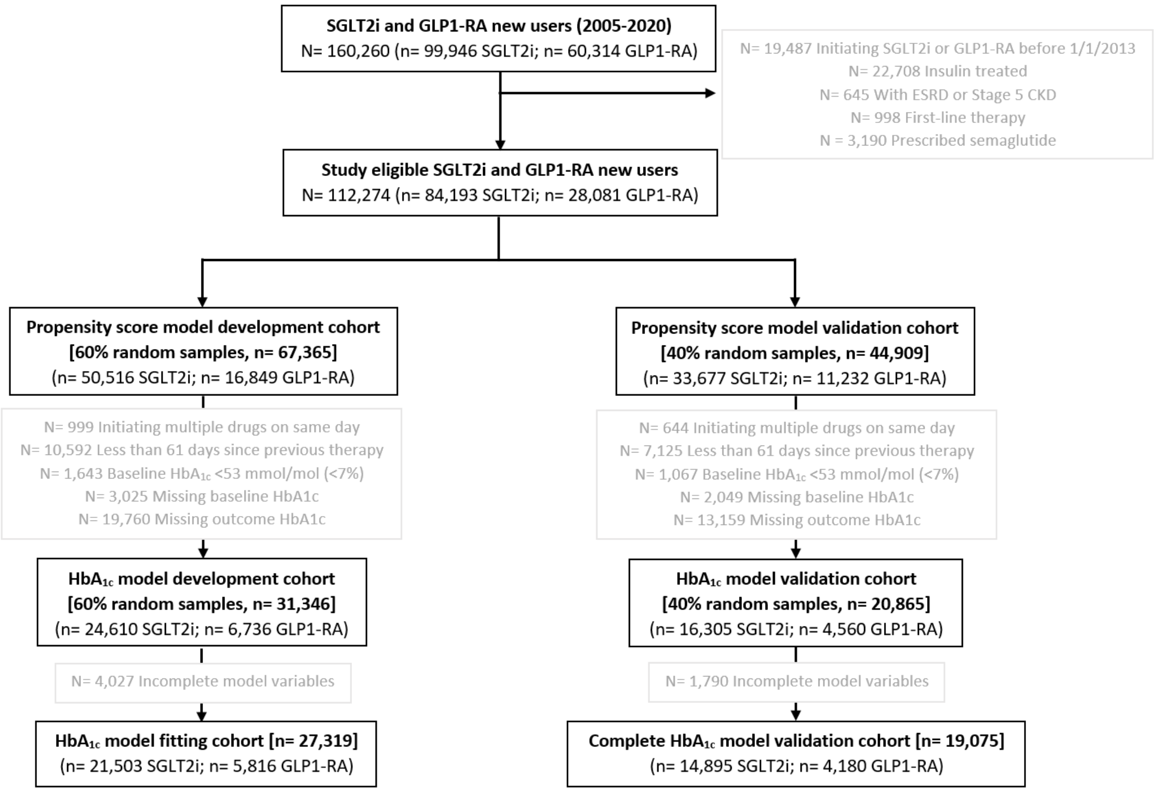

Baseline HbA_{1c} is defined as the closest HbA_{1c} to drug initiation in the previous 6 months. Other biomarkers were defined as the closest measure to drug initiation in the previous 2 years.

ESM Fig. 2: CPRD patient flowchart and inclusion criteria for the analysis of additional outcomes.

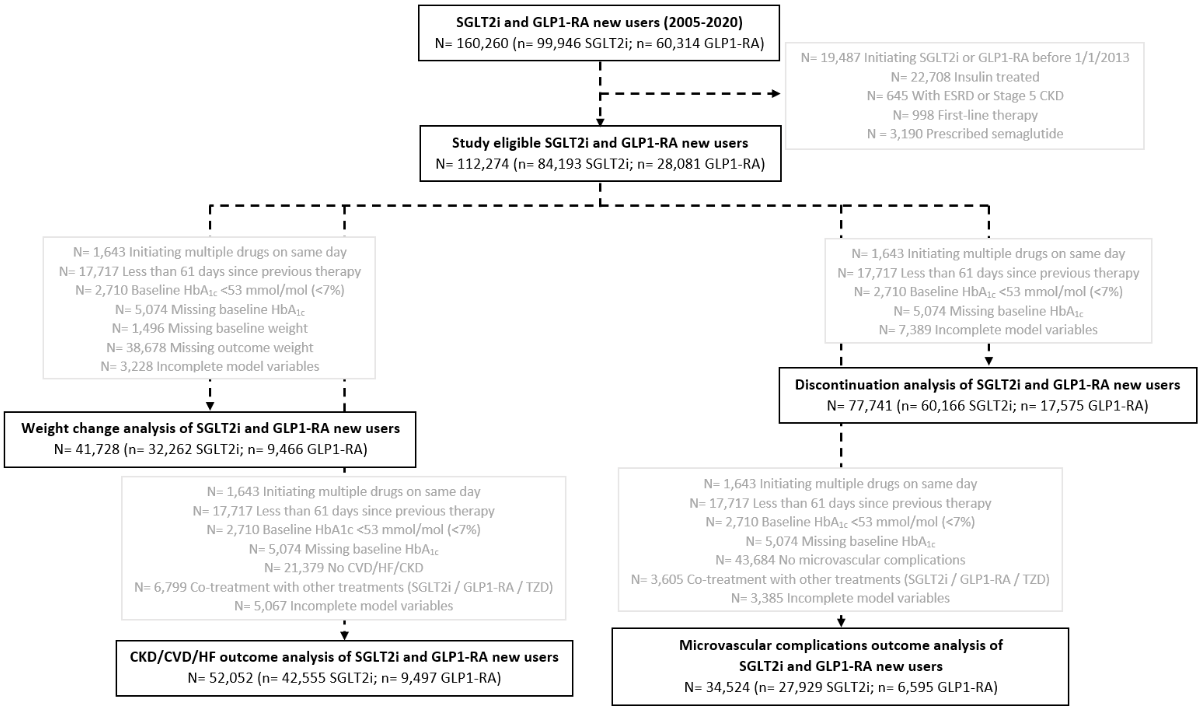

ESM Fig. 3: Variables selected for the prognostic (factors predictive of HbA_{1c} response to SGLT2i therapy) component (A) and the moderator (factors predictive of differential HbA_{1c} response with GLP1-RA compared to SGLT2i therapies) component (B) of the model.

Posterior inclusion proportions correspond to the average proportion of times each predictor is chosen as a splitting rule divided by the total number of splitting rules appearing in the model component. The threshold used corresponds to one divided by the number of variables used in the model times 100.

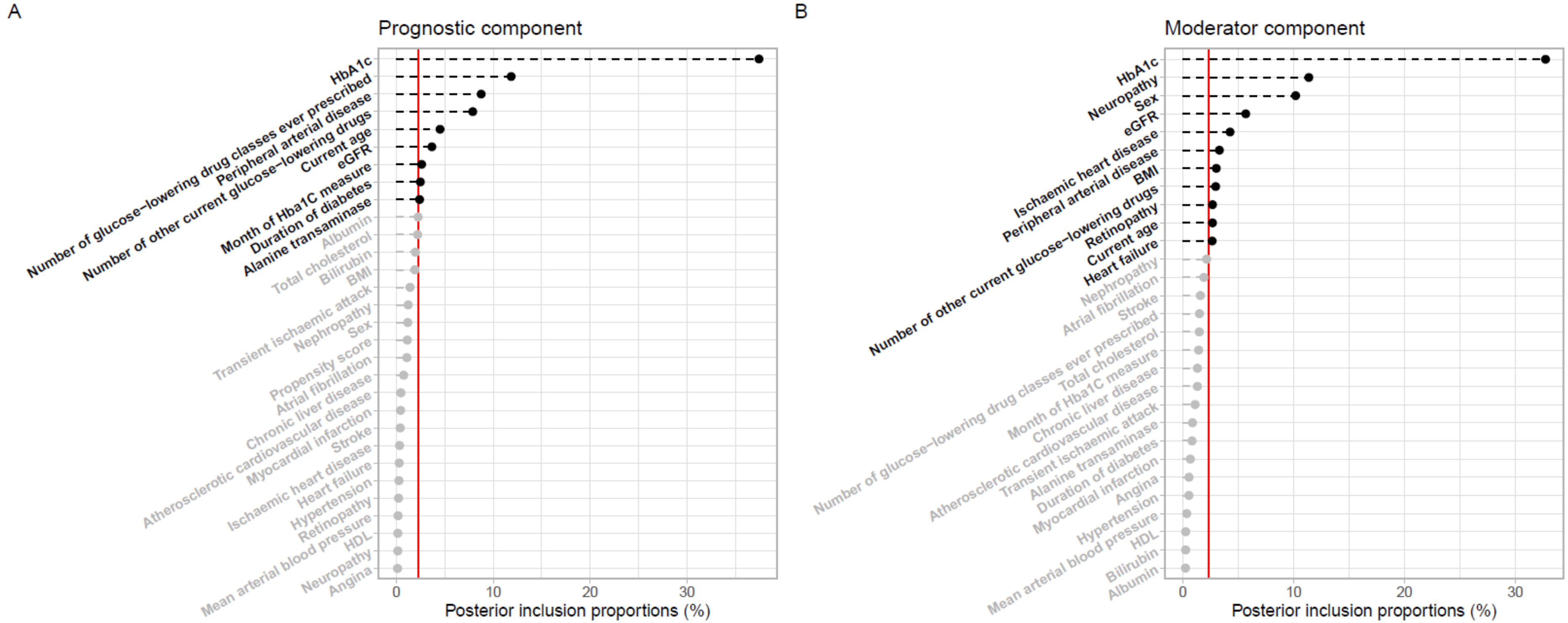

ESM Fig. 4: Comparison of predicted outcome HbA_{1c} and predicted conditional average treatment effect (CATE) estimates from a sparse Bayesian Causal Forest (BCF) model and a BCF model with different sets of variables.
Sparse BCF is fitted with all candidate predictors, and BCF is fitted with the selected variables from variable selection (see Table 2). (A.1) and (A.2) show predictions of outcomes HbA_{1c} under both models and a histogram of the predicted HbA_{1c} differences, respectively, and (B.1) and (B.2) are analogous but for the predicted CATE estimates.

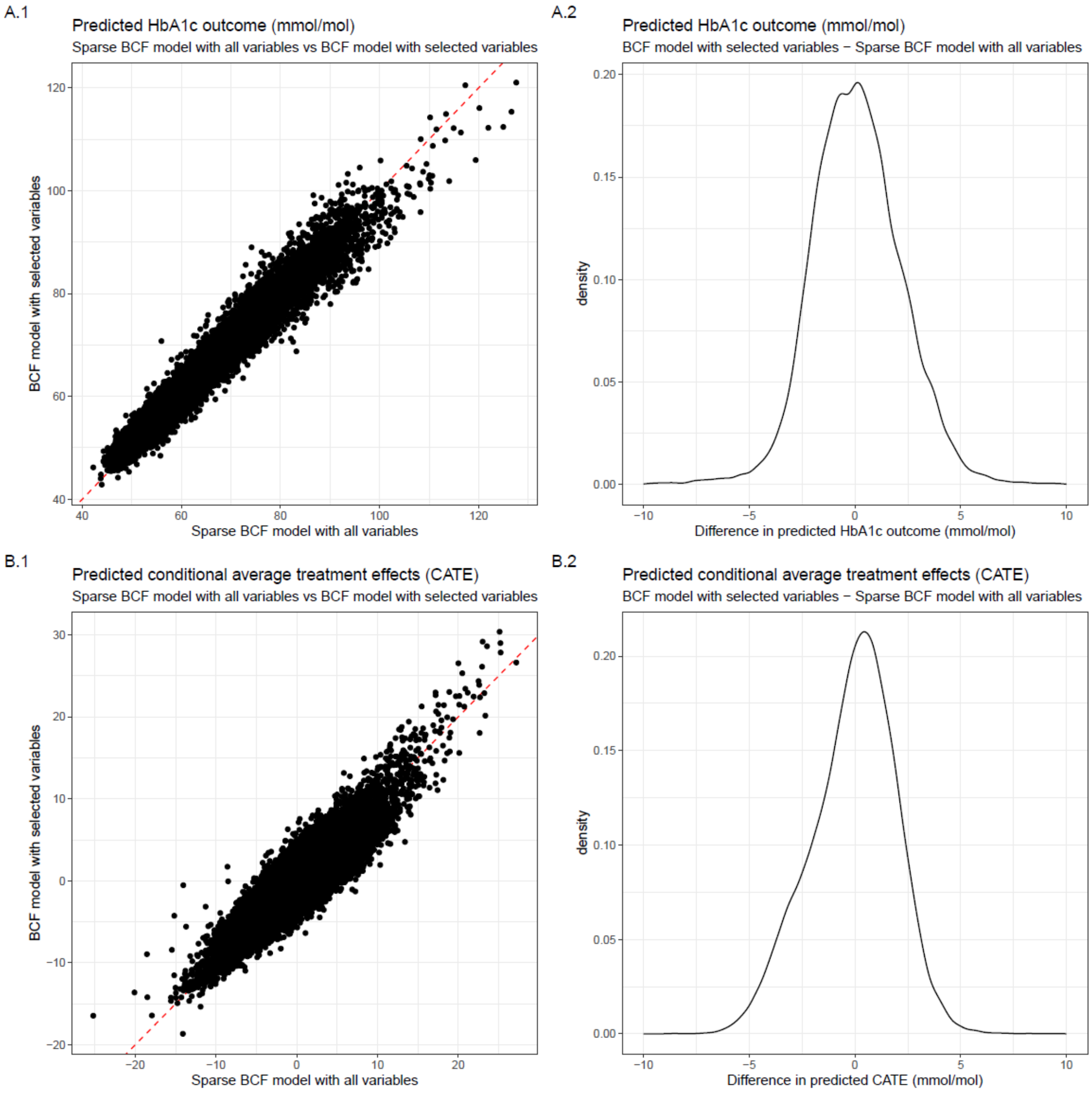

ESM Fig. 5: Comparison of predicted outcome HbA_{1c} and predicted conditional average treatment effect (CATE) estimates from two Bayesian Causal Forest (BCF) models with and without including propensity scores in the development cohort.

(A.1) and (A.2) show predictions of outcome HbA_{1c} under both models and a histogram of the predicted HbA_{1c} differences, respectively, and (B.1) and (B.2) are analogous but for the predicted CATE estimates.

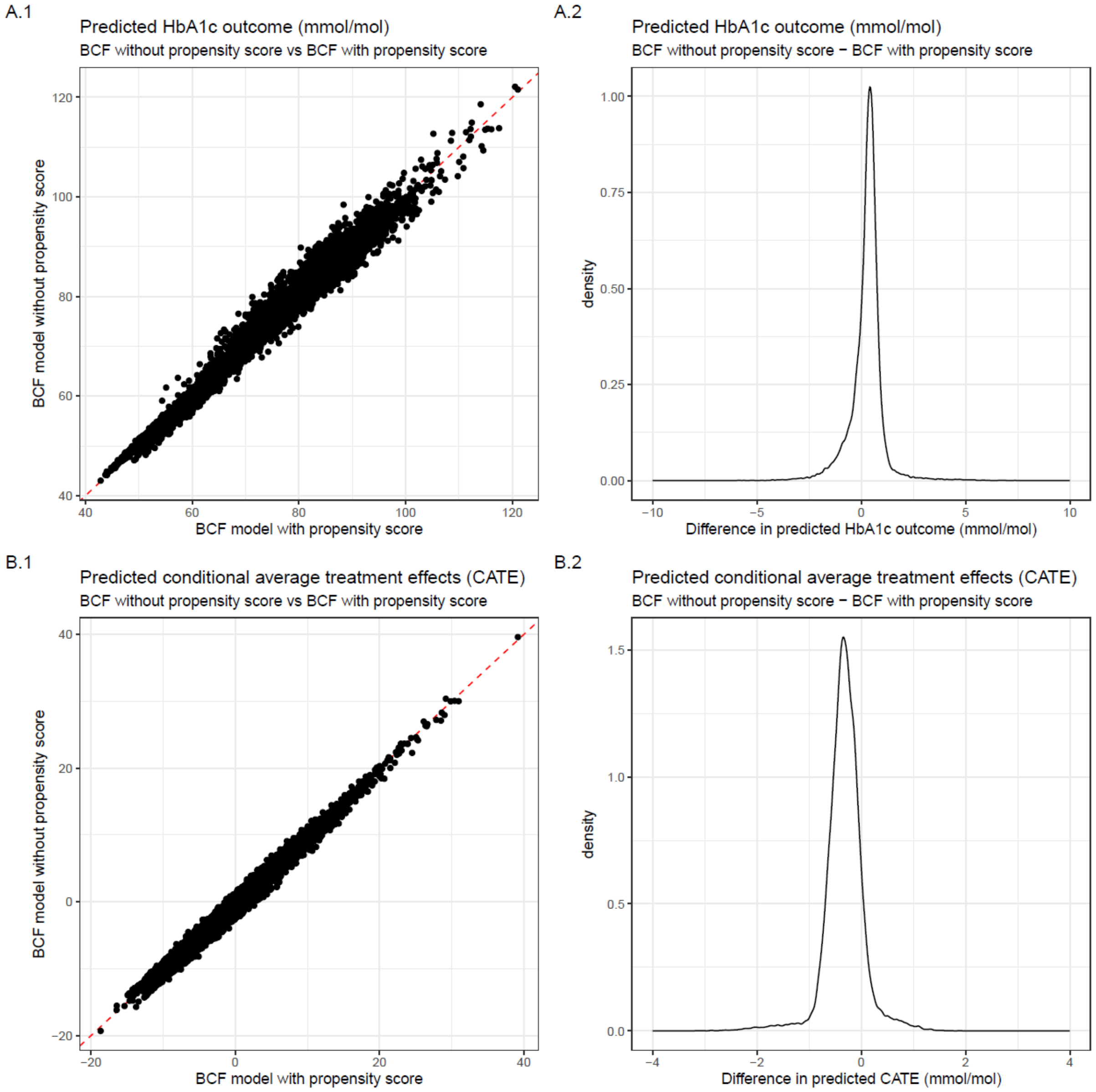

ESM Fig. 6: Variable selection plot for the propensity score model — identifying the most important predictors.
This plot presents the variable selection results of a propensity score model. The green lines represent the threshold levels determined from permutation distributions that must be exceeded for a variable to be selected by the model. The plotted points indicate the variable inclusion proportions for the observed data in the propensity score model. The solid dots represent the variables that are included in the model because their observed values exceed the corresponding green bars, while the open dots represent the variables that are not included in the model because their observed values fall below the threshold.

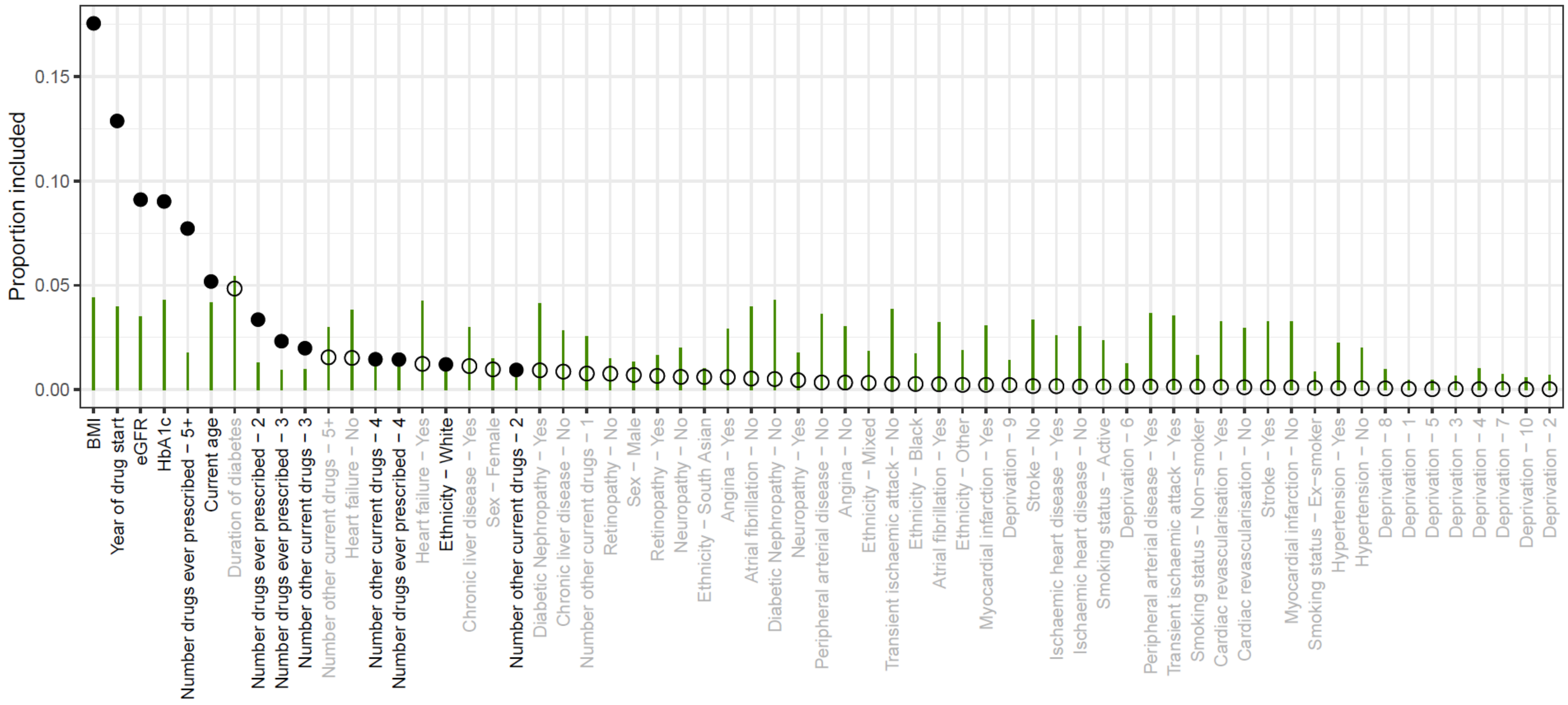

ESM Fig. 7: Received operating characteristic (ROC) and precision-recall curves of the propensity score model developed in the development cohort and validated in hold-out validation data.

The ROC curves (1) see similar performance in the development (A) and validation (B) cohorts, showing the model is performing well in correctly identifying when SGLT2i and GLP-1RA are indicated. Precision-recall curves (2) plot the proportion of true positives among all predicted positives, against the proportion of true positives among all actual positives. The precision-recall curves see similar performance in the development and validation cohorts, showing that the model can accurately identify positive instances (GLP-1RA) with minimal false positives for a proportion of thresholds.

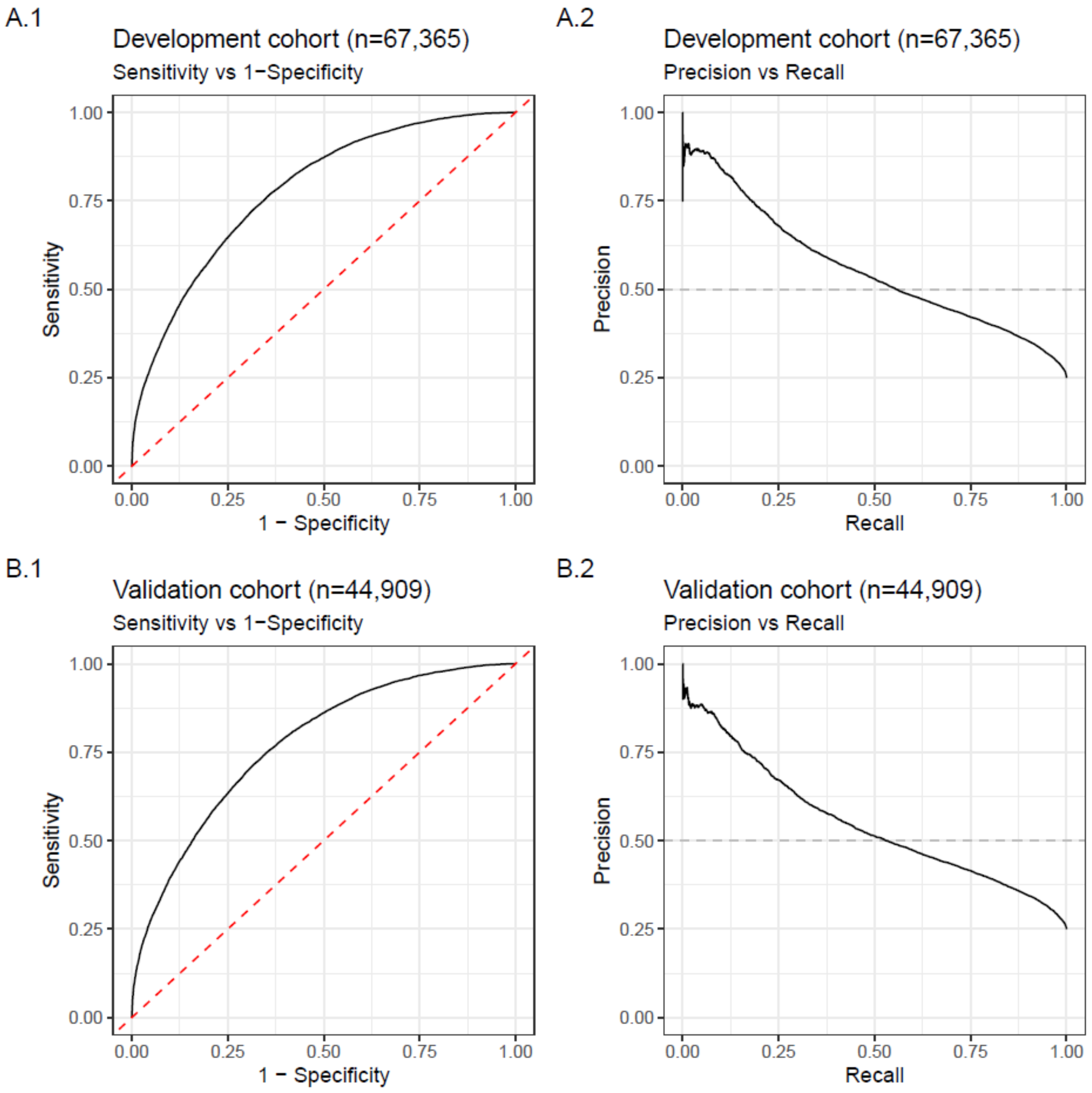

ESM Fig. 8: Calibration plots of predicted conditional treatment effect (CATE) estimates using propensity score matching.

(A) Calibration plots using unadjusted estimates of average treatment effects for each decile of predicted conditional average treatment effects in propensity score matched individuals of the development (A.1) and validation (A.2) cohorts. (B) Calibration plots using estimates adjusted for all variables used in the treatment selection model, in propensity score matched individuals of the development (B.1) and validation (B.2) cohorts.

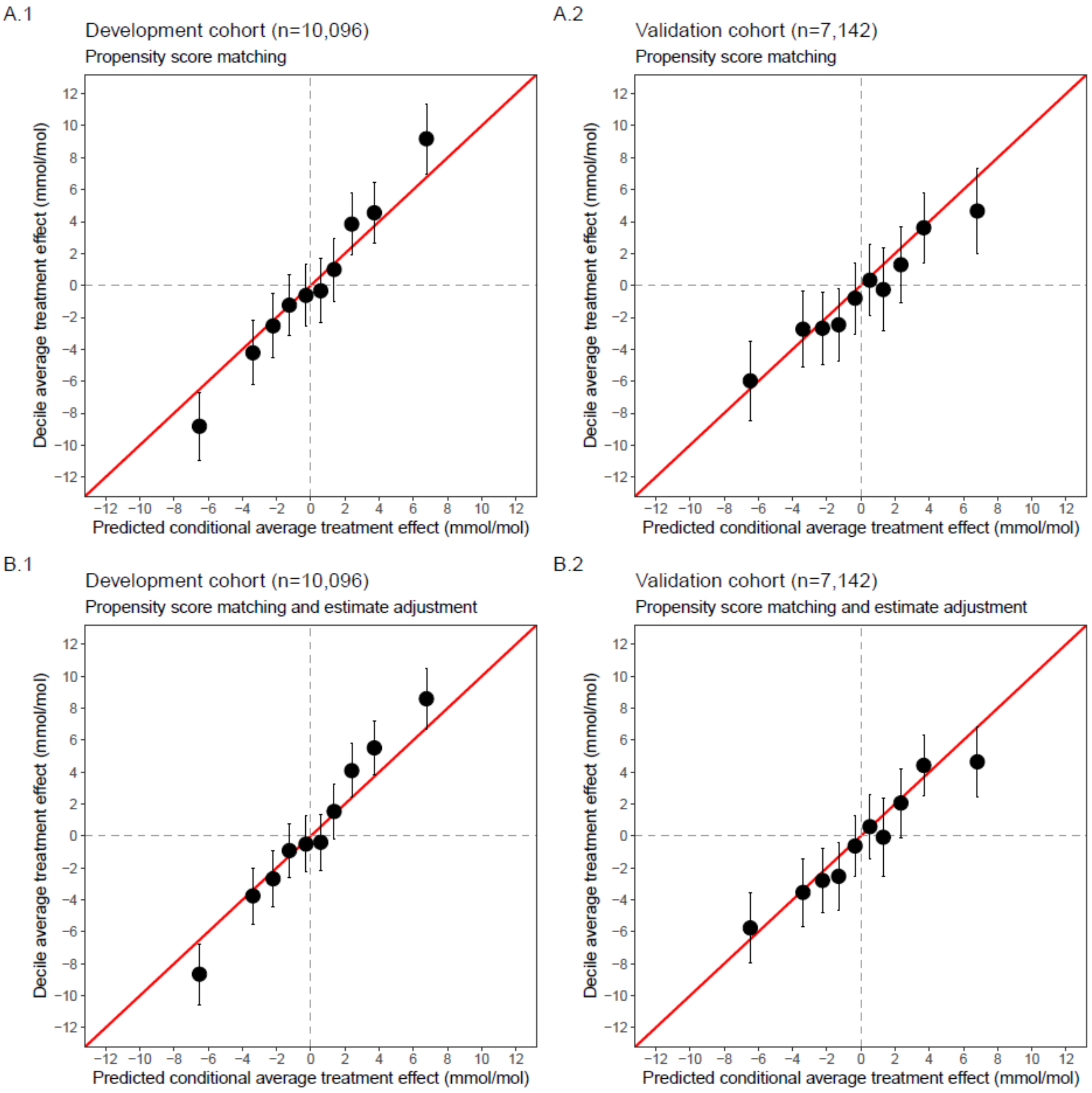

ESM Fig. 9: Model interpretability plots

(x A) Relative variable importance for clinical features predicting differential treatment effects (best linear projection of BCF model; see Methods). (B) Distribution of conditional average treatment effect (CATE) estimates for SGLT2i vs. GLP1-RA, by sex. (C) Predicted treatment effects for all differential clinical features, with individuals stratified into quintiles for continuous variables, and black lines corresponding to the median of the stratified group. All estimates are for the overall study population, n= 46,394.

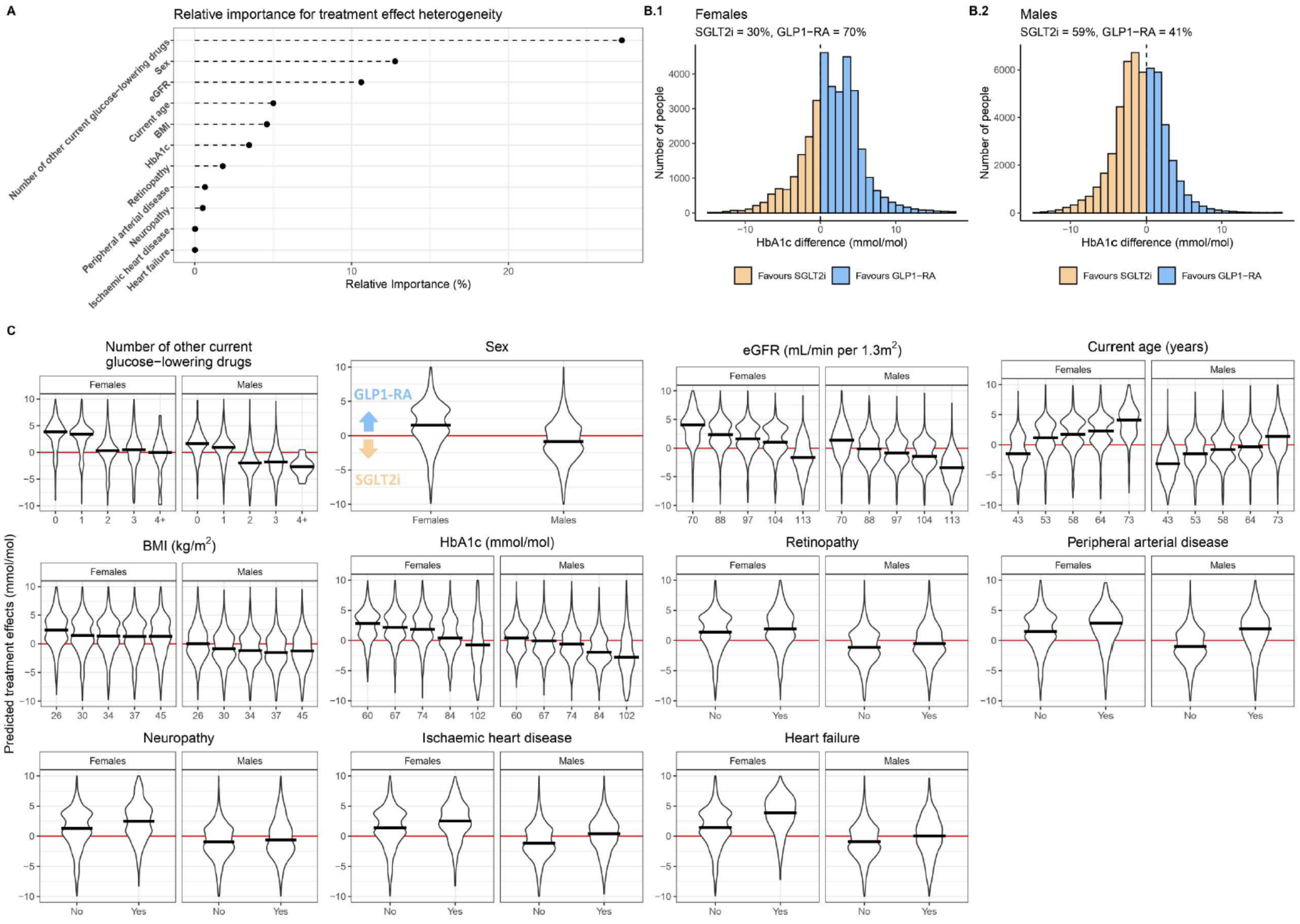

ESM Fig. 10: Differential HbA_{1c} treatment effects in individuals of white ethnicity and people of colour

Adjusted ATE estimates within subgroups defined by clinically meaningful CATE thresholds (SGLT2i benefit >5, 3-5 and 0-3 mmol/mol, GLP1-RA benefit >5, 3-5 and 0-3 mmol/mol). Negative values reflect a predicted glucose-lowering treatment benefit with SGLT2i, and positive values reflect a predicted treatment benefit with GLP1-RA. The people of colour subgroup is a composite of major UK self-reported ethnicity groups: Black, South Asian, Mixed and Other. Individuals without a recorded ethnicity were excluded (n=932).

Overall population (n=45,462)	White	People of Colour
Predicted HbA1c benefit on SGLT2i		
>5 mmol/mol	2636	775
3−5 mmol/mol	3576	956
0−3 mmol/mol	11107	2813
Predicted HbA1c benefit on GLP1−RA		
0−3 mmol/mol	11735	2650
3−5 mmol/mol	4930	998
>5 mmol/mol	2799	487
Average treatment effect	36783	8679

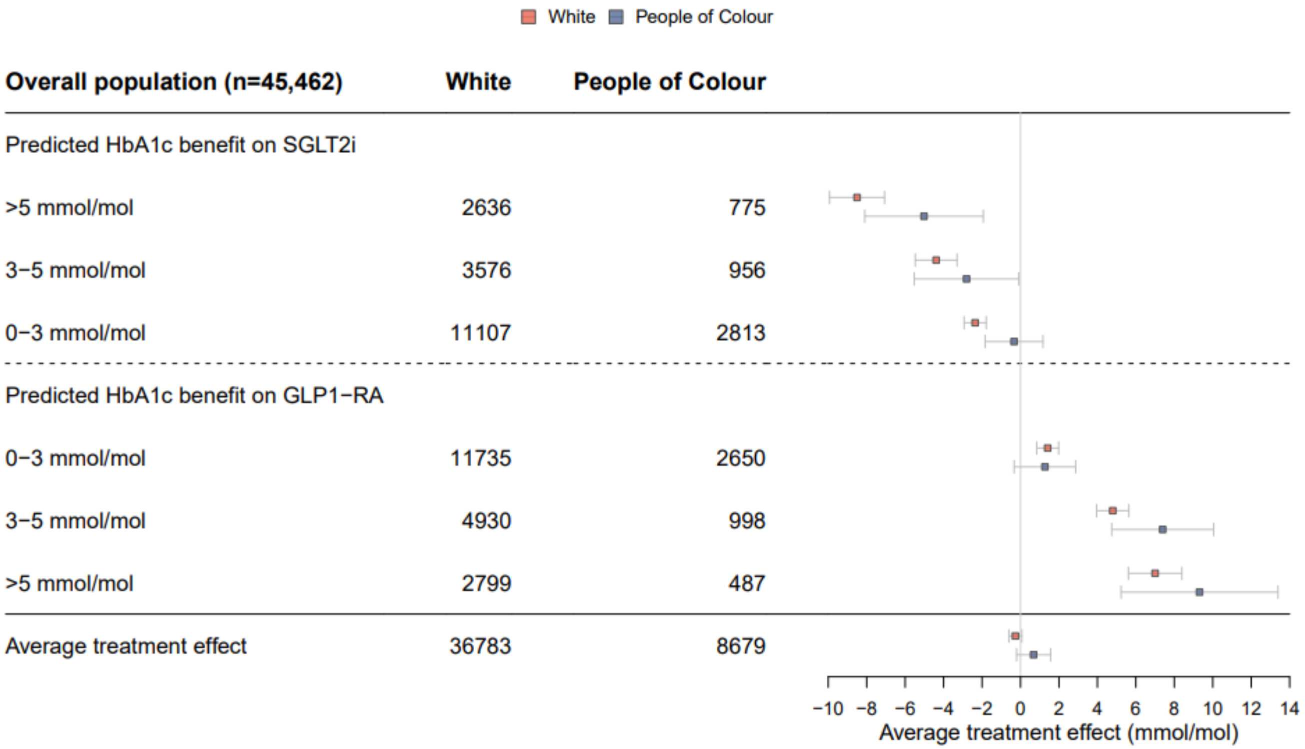

ESM Fig. 11: Calibration plots of predicted conditional individualised treatment effects in the validation cohort for those with and without cardiovascular disease (CVD).

Calibration in those with (A) and without (B) CVD in the hold-out validation cohort. Estimates are adjusted with all the variables used in the treatment selection model.

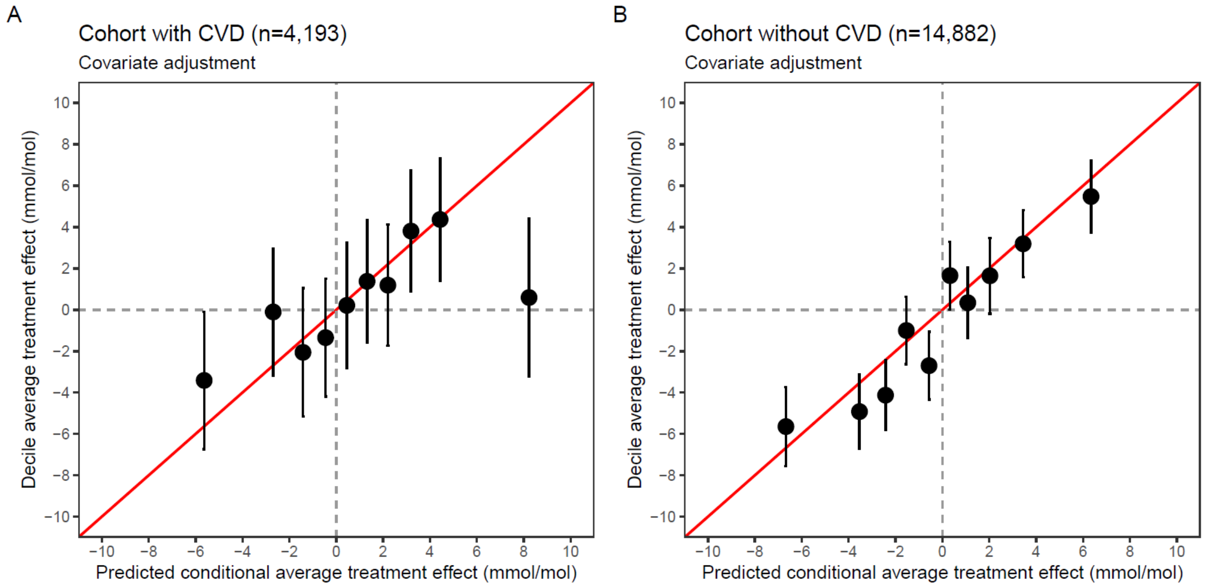

ESM Fig. 12: Relative risk of developing new onset chronic kidney disease (CKD) over 5 years, across subgroups defined by clinical cut-offs of predicted treatment benefit.

Chronic kidney disease (CKD) is defined by a drop of 40% in eGFR or reaching CKD – stage 5. Negative hazard ratio values correspond to a reduced risk of CKD on SGLT2-inhibitor treatment, and positive hazard ratio values correspond to a reduced risk of CKD on GLP-1 receptor agonist treatment. Estimates are split into three approaches: (A) all individuals with estimates adjusted for all the variables used in the treatment selection model; (B) propensity score matched individuals with unadjusted estimates; (C) propensity score matched individuals with estimates adjusted for all the variables used in the treatment selection model.

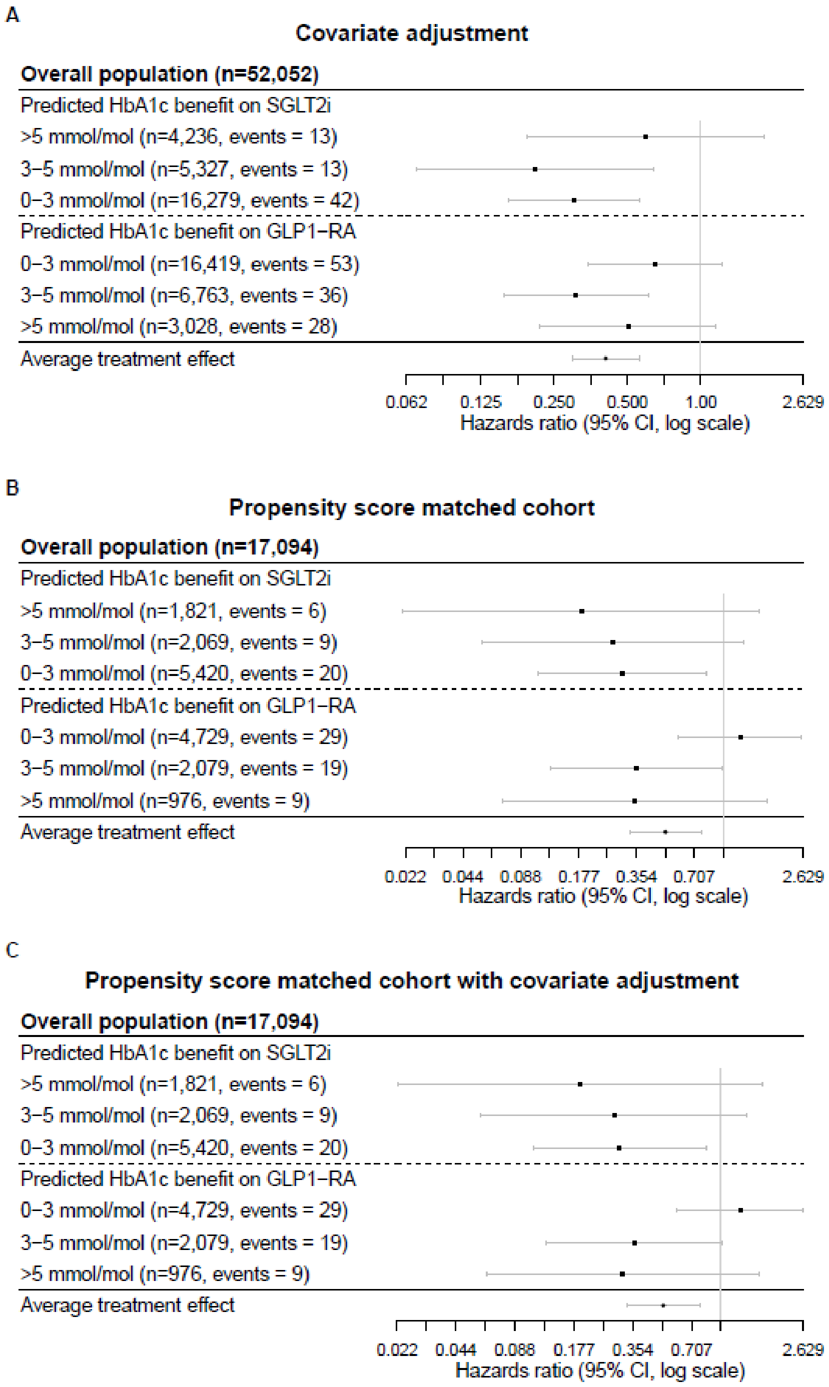

ESM Fig. 13: Short-term and long-term clinical outcomes, across subgroups defined by clinical cut-offs of predicted treatment benefit, in propensity score matched cohorts

Replication of Fig. 5 in propensity score matched individuals and without covariate adjustment. Estimates of short-term (A) and long-term (B) outcomes (GLP1-RA baseline group) are calculated with propensity score matched individuals. (A.1) 12-month predicted HbA_{1c} change on each treatment. (A.2) 12-month predicted weight change on each treatment. (A.3) 6-month risk of discontinuation on each treatment. (B.1) 5-year risk of developing microvascular complications. (B.2) 5-year of developing major adverse cardiovascular events (MACE). (B.3) 5-year risk of heart failure.

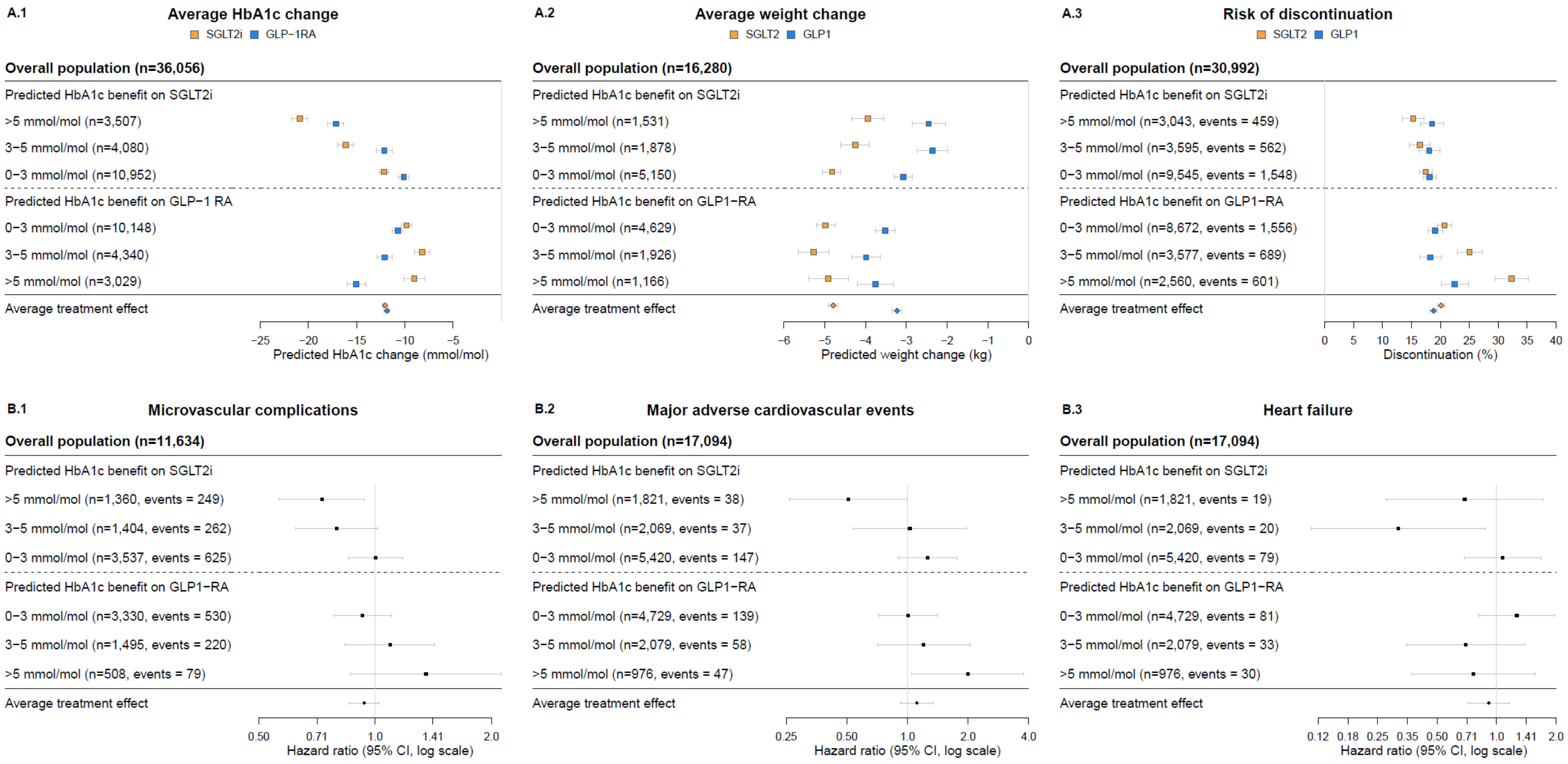

ESM Fig. 14: Short-term and long-term clinical outcomes, across subgroups defined by clinical cut-offs of predicted treatment benefit, in propensity score matched cohort with additional covariate adjustment.

Replication of Fig. 5 in propensity score matched individuals. Estimates of short-term (A) and long-term (B) outcomes (GLP1-RA baseline group) are calculated with propensity score matched individuals and adjusted with all the variables used in the treatment selection model. (A.1) 12-month predicted HbA_{1c} change on each treatment. (A.2) 12-month predicted weight change on each treatment. (A.3) 6-month risk of discontinuation on each treatment. (B.1) 5-year risk of developing microvascular complications. (B.2) 5-year of developing major adverse cardiovascular events (MACE). (B.3) 5-year risk of heart failure.

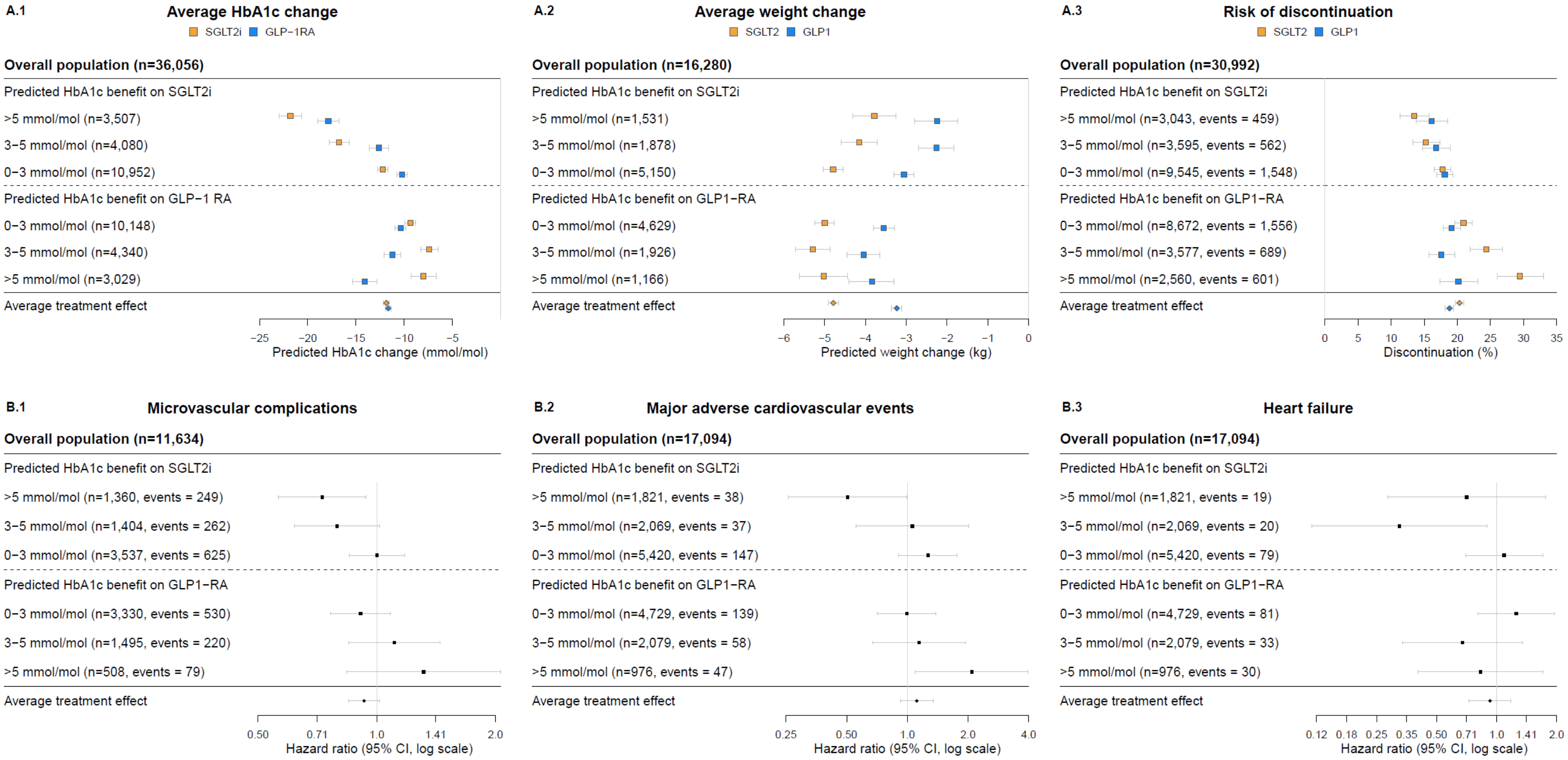

ESM Fig. 15: Evaluation of concordance between estimates of predicted HbA_{1c} outcome with SGLT2i from our model and a recently published SGLT2i-DPP4-inhibitor treatment selection model[13].

82,933 eligible individuals from the overall study population fulfilling inclusion criteria for both studies. (A) Comparison of outcome HbA_{1c} predictions for all patients from both models. The red dashed line corresponds to a theoretical equality between both models. The blue line corresponds to the trend line of the predictions. (B) Histogram of residual difference between HbA_{1c} predictions (mmol/mol) of both models.

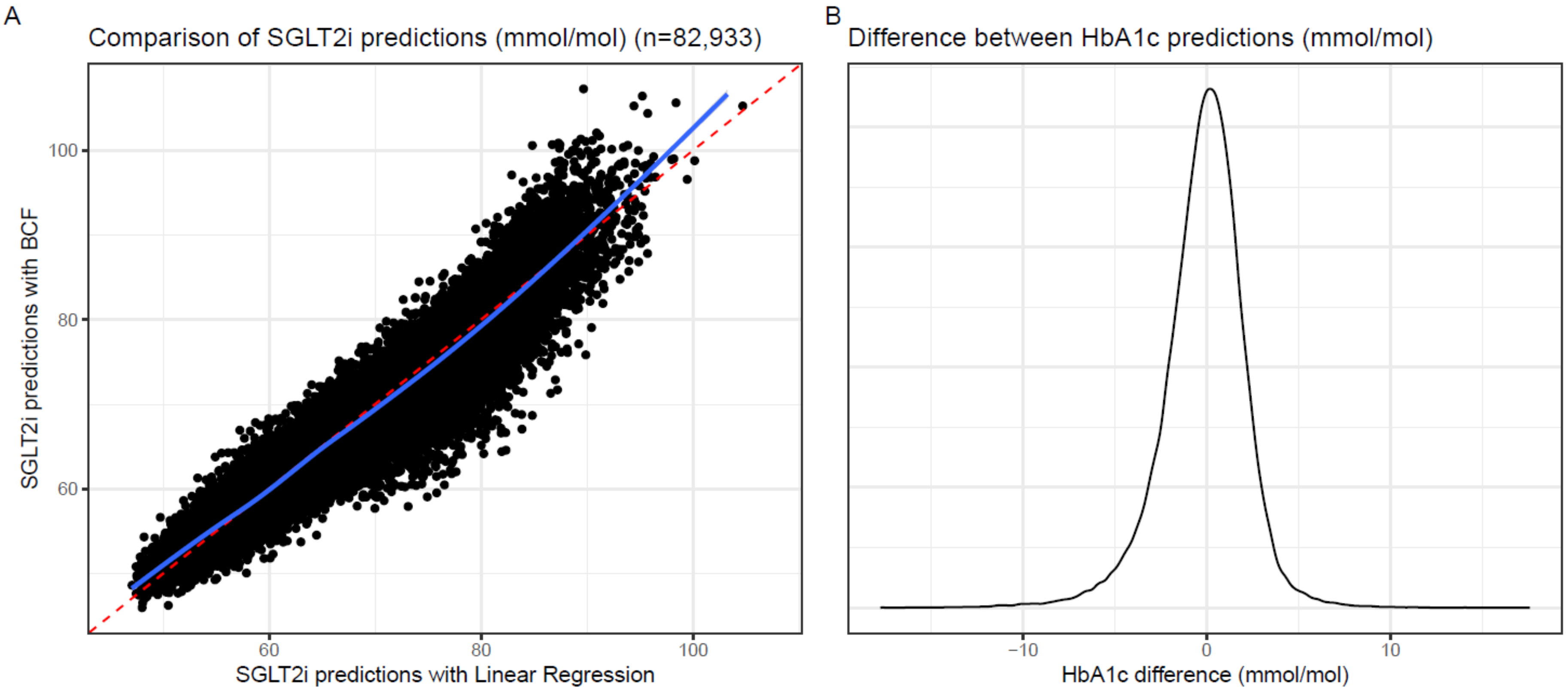

Section/Topic	Item		Checklist Item	Page
**Title and abstract**				
Title	1	D;V	Identify the study as developing and/or validating a multivariable prediction model, the target population, and the outcome to be predicted.	1
Abstract	2	D;V	Provide a summary of objectives, study design, setting, participants, sample size, predictors, outcome, statistical analysis, results, and conclusions.	2
**Introduction**				
Background and objectives	3a	D;V	Explain the medical context (including whether diagnostic or prognostic) and rationale for developing or validating the multivariable prediction model, including references to existing models.	4
	3b	D;V	Specify the objectives, including whether the study describes the development or validation of the model or both.	4
**Methods**				
Source of data	4a	D;V	Describe the study design or source of data (e.g., randomized trial, cohort, or registry data), separately for the development and validation data sets, if applicable.	5
	4b	D;V	Specify the key study dates, including start of accrual; end of accrual; and, if applicable, end of follow-up.	5
Participants	5a	D;V	Specify key elements of the study setting (e.g., primary care, secondary care, general population) including number and location of centres.	5
	5b	D;V	Describe eligibility criteria for participants.	5
	5c	D;V	Give details of treatments received, if relevant.	5
Outcome	6a	D;V	Clearly define the outcome that is predicted by the prediction model, including how and when assessed.	5
	6b	D;V	Report any actions to blind assessment of the outcome to be predicted.	7
Predictors	7a	D;V	Clearly define all predictors used in developing or validating the multivariable prediction model, including how and when they were measured.	6
	7b	D;V	Report any actions to blind assessment of predictors for the outcome and other predictors.	6
Sample size	8	D;V	Explain how the study size was arrived at.	5
Missing data	9	D;V	Describe how missing data were handled (e.g., complete-case analysis, single imputation, multiple imputation) with details of any imputation method.	5,6,7
Statistical analysis methods	10a	D	Describe how predictors were handled in the analyses.	6,7
	10b	D	Specify type of model, all model-building procedures (including any predictor selection), and method for internal validation.	6,7,8,9
	10c	V	For validation, describe how the predictions were calculated.	7,8,9
	10d	D;V	Specify all measures used to assess model performance and, if relevant, to compare multiple models.	7,8,9
	10e	V	Describe any model updating (e.g., recalibration) arising from the validation, if done.	-
Risk groups	11	D;V	Provide details on how risk groups were created, if done.	-
Development vs. validation	12	V	For validation, identify any differences from the development data in setting, eligibility criteria, outcome, and predictors.	5
**Results**				
Participants	13a	D;V	Describe the flow of participants through the study, including the number of participants with and without the outcome and, if applicable, a summary of the follow-up time. A diagram may be helpful.	10
	13b	D;V	Describe the characteristics of the participants (basic demographics, clinical features, available predictors), including the number of participants with missing data for predictors and outcome.	10
	13c	V	For validation, show a comparison with the development data of the distribution of important variables (demographics, predictors and outcome).	10
Model development	14a	D	Specify the number of participants and outcome events in each analysis.	10
	14b	D	If done, report the unadjusted association between each candidate predictor and outcome.	-
Model specification	15a	D	Present the full prediction model to allow predictions for individuals (i.e., all regression coefficients, and model intercept or baseline survival at a given time point).	-
	15b	D	Explain how to the use the prediction model.	10,11
Model performance	16	D;V	Report performance measures (with CIs) for the prediction model.	11,12
Model-updating	17	V	If done, report the results from any model updating (i.e., model specification, model performance).	11,12,13
**Discussion**				
Limitations	18	D;V	Discuss any limitations of the study (such as nonrepresentative sample, few events per predictor, missing data).	14,15
Interpretation	19a	V	For validation, discuss the results with reference to performance in the development data, and any other validation data.	14,15
	19b	D;V	Give an overall interpretation of the results, considering objectives, limitations, results from similar studies, and other relevant evidence.	14,15
Implications	20	D;V	Discuss the potential clinical use of the model and implications for future research.	14,15
**Other information**				
Supplementary information	21	D;V	Provide information about the availability of supplementary resources, such as study protocol, Web calculator, and data sets.	13,17
Funding	22	D;V	Give the source of funding and the role of the funders for the present study.	17

*Items relevant only to the development of a prediction model are denoted by D, items relating solely to a validation of a prediction model are denoted by V, and items relating to both are denoted D;V. We recommend using the TRIPOD Checklist in conjunction with the TRIPOD Explanation and Elaboration document.

References

[1] Caron A, Baio G, Manopoulou I (2021) Shrinkage Bayesian causal forests for heterogeneous treatment effects estimation. Journal of Computational and Graphical Statistics 31(4): 1202-1214
[2] Caron A (2020) SparseBCF: sparse Bayesian causal forest for heterogeneous treatment.
[3] Hahn PR, Murray JS, Carvalho CM (2020) Bayesian regression tree models for causal inference: regularization, confounding, and heterogeneous effects (with discussion). Bayesian Analysis 15(3): 965-1056
[4] Chipman HA, George EI, McCulloch RE (2010) BART: Bayesian additive regression trees.
[5] Adam Kapelner JB (2013) bartMachine: Machine Learning with Bayesian Additive Regression Trees. arXiv
[6] Hahn PR, Murray JS, Carvalho CM (2020) Bayesian regression tree models for causal inference: regularization, confounding, and heterogeneous effects (with discussion). Bayesian Analysis: 15(13): 965-1056
[7] Kapelner A, Bleich J (2013) bartMachine: Machine Learning with Bayesian Additive Regression Trees. arXiv
[8] Bleich J, Kapelner A, George EI, Jensen ST (2014) Variable selection for BART: an application to gene regulation. The Annals of Applied Statistics 8(3): 1750-1781
[9] Gelman A, Rubin DB (1992) Inference from iterative simulation using multiple sequences. Statistical Science 7(4): 457-472
[10] Tibshirani J, Athey S, Sverdrup E, Wager S (2023) grf: Generalized Random Forests. In:
[11] Cui Y, Kosorok MR, Sverdrup E, Wager S, Zhu R (2023) Estimating heterogeneous treatment effects with right-censored data via causal survival forests. Journal of the Royal Statistical Society Series B: Statistical Methodology 85(2): 179-211
[12] Caliendo M, Scheel-Kopeinig S (2008) Some practical guidance for the implementation of propensity score matching. Journal of Economic Surveys 22(1): 31-72
[13] Dennis JM, Young KG, McGovern AP, et al. (2022) Development of a treatment selection algorithm for SGLT2 and DPP-4 inhibitor therapies in people with type 2 diabetes: a retrospective cohort study. The Lancet Digital Health: 4(12): 873-883

PRIBA Study Group

Lead Centre:

Royal Devon and Exeter NHS Foundation Trust/University of Exeter: Anita Hill, Rob Bolt, Jane Stewart, Bridget Knight, Tim McDonald, Beverley Shields, Angus Jones, Andrew Hattersley, Gayle Githens-Mazer, Tina Sanders, Kirsty Wensley

NIHR Clinical Research Network:

Ipswich Hospital NHS Trust: Gerry Rayman, Sue Hood, Jo Rosier, Jane Jiao, Debbie Simmonds, Caroline Calver
North Bristol Hospitals NHS Trust: Andrew Johnson, Sharon Tovey, Jade Bennet, Dafydd Wilson Evans, Philippa Lamb, Hilary Holloway, B Moore
Northampton General Hospital NHS Trust: Charles Fox, Kathy Hall, L James, C Smith
Northern Devon Healthcare NHS Trust: Alastair Watt, Geraldine Belcher, Amanda Skinner
Oxford Centre for Diabetes, Endocrinology and Metabolism: Steve Gough, Judy MacDonald, Lynne Nairn, Sue Rous
Plymouth Hospitals NHS Trust: Ann Millward, Margaret Blackmore, Migaila Watt
Portsmouth Hospitals NHS Trust: Mike Cummings, Sharon Allard, Elaine Hallett, Jane Rowney
Royal Bournemouth and Christchurch Hospitals NHS Foundation Trust: David Kerr, Patricia Sanders, Carina Vickers
Royal Cornwall Hospitals NHS Foundation Trust: Steve Creely, Duncan Browne, Helen Chenoweth, Terri Chant, Sue Durkin
Royal Stoke University Hospital North Midlands: Ellen Hodgson, Gemma Reddell, Loretta Barnett, Jane Deleaney
South Devon Healthcare NHS Foundation Trust: Richard Paisey, Sue Bunce, Dawn Tomlinson, Mary Costello
South Warwickshire NHS Foundation Trust: Peter Horrocks, Penny Parsons, Alex Smith
Surrey and Sussex Healthcare NHS Trust: James Clark, Tracey Shewan, Louise Nimako
Taunton and Somerset NHS Foundation Trust: Rob Andrews, Catherine Thompson, Donna Archer
West Hertfordshire Hospitals NHS Trust: Thomas Galliford, Elaine Walker, Lynn Curry, Sindi Masuka, Cathy Constantin
Yeovil District Hospital NHS Foundation Trust: Seshadri Pramodh, Linda Balian, James Gibbons, Claire Buckley

MASTERMIND consortium

Prof Andrew Hattersley[1], Prof Ewan Pearson[2], Dr Angus Jones[1], Dr Beverley Shields[1], Dr John Dennis[1], Dr Lauren Rodgers[1], Prof William Henley[1], Prof Timothy McDonald[1], Prof Michael Weedon[1], Prof Nicky Britten[1], Catherine Angwin[1], Dr Naveed Sattar[3], Dr Robert Lindsay[3], Prof Christopher Jennison[4], Prof Mark Walker[5], Prof Kennedy Cruickshank[6], Dr Salim Janmohamed[7], Prof Christopher Hyde[1], Prof Rury Holman[8], Prof Andrew Farmer[8], Prof Alastair Gray[8], Prof Stephen Gough[8], Dr Olorunsola Agbaje[8], Dr Trevelyan McKinley[1], Dr Sebastian Vollmer[9], Dr Bilal Mateen[7], Prof William Hamilton[1], Dr Katie G. Young[1], Dr Pedro Cardoso[1], Dr Laura Güdemann[1]

[1] University of Exeter
[2] University of Dundee
[3] University of Glasgow
[4] University of Bath
[5] University of Newcastle
[6] Kings College London
[7] University College London
[8] University of Oxford
[9] University of Kaiserslautern
